# Multiple copies of a novel amphipathic α-helix forming segment in *Physcomitrella patens* dehydrin play a key role in abiotic stress mitigation

**DOI:** 10.1016/j.jbc.2021.100596

**Published:** 2021-03-26

**Authors:** Gouranga Upadhyaya, Arup Das, Chandradeep Basu, Tanushree Agarwal, Chandra Basak, Chandrima Chakraborty, Tanmoy Halder, Gautam Basu, Sudipta Ray

**Affiliations:** 1 Plant Functional Genomics Laboratory, Department of Botany, University of Calcutta, Kolkata, India; 2 Department of Biophysics, Bose Institute, Kolkata, India

**Keywords:** amphipathic α-helix, high-temperature stress, dehydrin–dehydrin interaction, protein aggregation, self-association, cell survivability, nuclear magnetic resonance (NMR), intrinsically disordered protein, poikilohydric plant, D-segment, BiFC, bimolecular fluorescence complementation, DHN, dehydrin, IDP, intrinsically disordered protein, LDH, lactate dehydrogenase, LEA, late embryogenesis abundant, MDA, malondialdehyde, NT, nontransformed, PpDHNA, *Physcomitrella patens* dehydrin, TFE, trifluoroethanol, VT, vector transformed

## Abstract

Plants use a diverse set of proteins to mitigate various abiotic stresses. The intrinsically disordered protein dehydrin is an important member of this repertoire of proteins, characterized by a canonical amphipathic K-segment. It can also contain other stress-mitigating noncanonical segments—a likely reflection of the extremely diverse nature of abiotic stress encountered by plants. Among plants, the poikilohydric mosses have no inbuilt mechanism to prevent desiccation and therefore are likely to contain unique noncanonical stress-responsive motifs in their dehydrins. Here we report the recurring occurrence of a novel amphipathic helix-forming segment (D-segment: EGφφD(R/K)AKDAφ, where φ represents a hydrophobic residue) in *Physcomitrella patens* dehydrin (PpDHNA), a poikilohydric moss. NMR and CD spectroscopic experiments demonstrated the helix-forming tendency of the D-segment, with the shuffled D-segment as control. PpDHNA activity was shown to be size as well as D-segment dependent from *in vitro*, *in vivo*, and *in planta* studies using PpDHNA and various deletion mutants. Bimolecular fluorescence complementation studies showed that D-segment-mediated PpDHNA self-association is a requirement for stress abatement. The D-segment was also found to occur in two rehydrin proteins from *Syntrichia ruralis*, another poikilohydric plant like *P. patens*. Multiple occurrences of the D-segment in poikilohydric plant dehydrins/rehydrins, along with the experimental demonstration of the role of D-segment in stress abatement, implies that the D-segment mediates unique resurrection strategies, which may be employed by plant dehydrins that are capable of mitigating extreme stress.

Plants have developed multiple adaptive mechanisms to survive against various abiotic stresses. These include anatomical changes, production of osmolytes, and overproduction of stress-combating proteins. Late embryogenesis abundant (LEA) proteins are a group of highly hydrophilic glycine-rich proteins that accumulate late during embryogenesis and also function under stress conditions (1). Among the members of LEA proteins, dehydrins (DHNs) constitute a group of intrinsically disordered proteins (IDPs) involved in combating stress conditions (2). A conserved feature of all DHNs, sequenced till date, is the presence of a C-terminal K-segment, containing a lysine-rich 15-residue sequence stretch (EKKGIM(E/D)KIKEKLPG). Some DHNs also contain one or more Y-segments [(V/T)D(E/Q)YGNP]. In addition, DHNs may also contain a serine-rich S-segment [LHRSGS_4-10_(E/D_3_)] and/or a less conserved Φ-segment (3). Based on the number and arrangement of the Y-, S-, and K-segments, DHNs are classified into different subclasses: Y_n_SK_n_, Y_n_K_n_, SK_n_, K_n_, and K_n_S (4). Recently, a new amino acid stretch, called the F-segment (DRGLFDFLGKK), was identified in SK_n_-type DHNs across all seed plants (5).

DHN is unstructured in aqueous environment, forming intermolecular hydrogen bonds with neighboring water molecules in addition to intramolecular hydrogen bonds (6). A decrease in the hydration status of DHN leads to conformational changes. The formation of amphipathic α-helices, owing to the presence of K-segments in DHN molecules, is well known (7, 8, 9). These changes have been found to occur maximally in the presence of phospholipid membranes. A recent study demonstrated that the conserved K-segment, present in *Arabidopsis thaliana* DHN Lti30 (a K_6_ DHN), which binds to the phospholipid membrane, is driven by electrostatics, where the disordered K-segments locally fold into α-helices on the membrane surface (10, 11). During abiotic stress conditions, when other cellular globular proteins tend to denature and aggregate, DHN, being an IDP, does not aggregate. Instead, it prevents other proteins from aggregation through macromolecular shielding (6, 12).

The amphipathic α-helical conformation of the K-segment is an important factor influencing the stress-protective activity of DHN. This is reflected in the fact that DHN partially loses its cryoprotective nature upon mutations in the hydrophobic amino acids of K-segments (13). A study on wheat DHNs (WZY2 and DHN5) highlighted the crucial role of the canonical K-segments in stress tolerance in *Escherichia coli* cells (14, 15). *Arabidopsis* DHNs (ERD10 and ERD14) are also known to exhibit protection against thermal aggregation *in vitro* (16). Elaborating on the details of the functional mechanism under heat stress, it was concluded that the protection was mainly achieved through protection of the cellular proteome owing to the chaperonic activity of ERD14 *via* multimer formation (17).

The chaperone-like property of DHNs against abiotic stress is thought to arise owing to multimeric bundle formation, mediated by the stress-induced amphipathic α-helices formed by the K-segments (18, 19). The bundle, in turn, might interact with other proteins and phospholipid membrane surfaces under low hydration status (9, 20, 21, 22). The shielding effect, therefore, helps DHN to prevent denaturation of other cellular proteins, caused primarily due to the loss of water layer that envelopes folded proteins (2). Multimeric DHNs with large hydrodynamic radii form molecular shields between neighboring protein molecules, thus preventing collisions between them. Size and degree of disorder are the two important factors that determine the stress-protective ability of DHNs (23). Of interest, homodimeric and heterodimeric interactions have been shown to occur in studies performed on transformed plants overexpressing DHNs (24).

Transgenic plants have been extensively used to study the abiotic stress tolerance property of DHNs. Till date, numerous reports have shown that DHNs protect plants from desiccation, cold, salinity, and oxidative damage (2, 25). More recently, tolerance against high-temperature stress has been reported in transformed tobacco plants overexpressing *Sorghum bicolor* DHN1 protein (26). A recent study on the spliced variant of *Vitis vinifera* DHN1a reported that the K-segment stretch might play an important role in drought resistance (27). However, *in planta* studies of the protective role of the amphipathic α-helices of DHN under abiotic stress conditions are scanty, although such studies might provide a detailed insight into the molecular mechanism of action of DHNs in plants.

*Physcomitrella patens*, a member of the moss family, is a model organism and an excellent system to study many important aspects of plant biology. Specifically, its ability to tolerate desiccation stress has attracted scientific attention across the globe (28). The single-cell-layer thick organism is known to survive in almost 90% to 95% of water loss conditions (29). This typical poikilohydric nature of *P. patens*, along with the associated resurrection mechanism, has established it as a model organism for studying the underlying mechanisms of abiotic stress adaptation. The major DHN from *P. patens* (PpDHNA) has already been demonstrated as a key stress-responsive protein, for its abiotic stress (osmotic and salt) tolerance ability (30, 31, 32). DHNA-targeted knockout in *P. patens* further established its contribution to cellular protection under osmotic stress (31).

In an earlier study we had reported that the Y_11_K-type DHN (PpDHNA) was able to protect lactate dehydrogenase (LDH) and could rescue tobacco plants overexpressing PpDHNA from drought stress (33). Of interest, earlier studies showed that PpDHNA renders an unusually high degree of stress protection. This compelled us to look carefully at the sequence of PpDHNA to identify the presence of any PpDHNA-specific sequence motif and, if present, establish their functional roles, if any. Here we report the presence of an amphipathic α-helix forming segment in PpDHNA that we term as the D-segment. The D-segment, with the sequence motif EGφφD(R/K)AKDAφ, where φ represents a hydrophobic residue, along with Y- and K-segments were analyzed for their role in rendering protection under high-temperature and desiccation stress. A number of deletion mutants of PpDHNA were generated and assayed for their protection ability under both *in vitro* and *in vivo* conditions. We show that the D-segments play a significant role for the observed abiotic stress mitigation by PpDHNA. We further demonstrated the presence of the D-segment in a stress-responsive protein in another poikilohydric plant *Syntrichia ruralis*. Finally, D-segment-mediated unique resurrection and stress abatement strategies employed by plant dehydrins are discussed.

## Results

### Sequence analysis of PpDHNA predicted stretches of amino acid sequences (D-segments) capable of amphipathic α-helix formation

**Figure 1:**
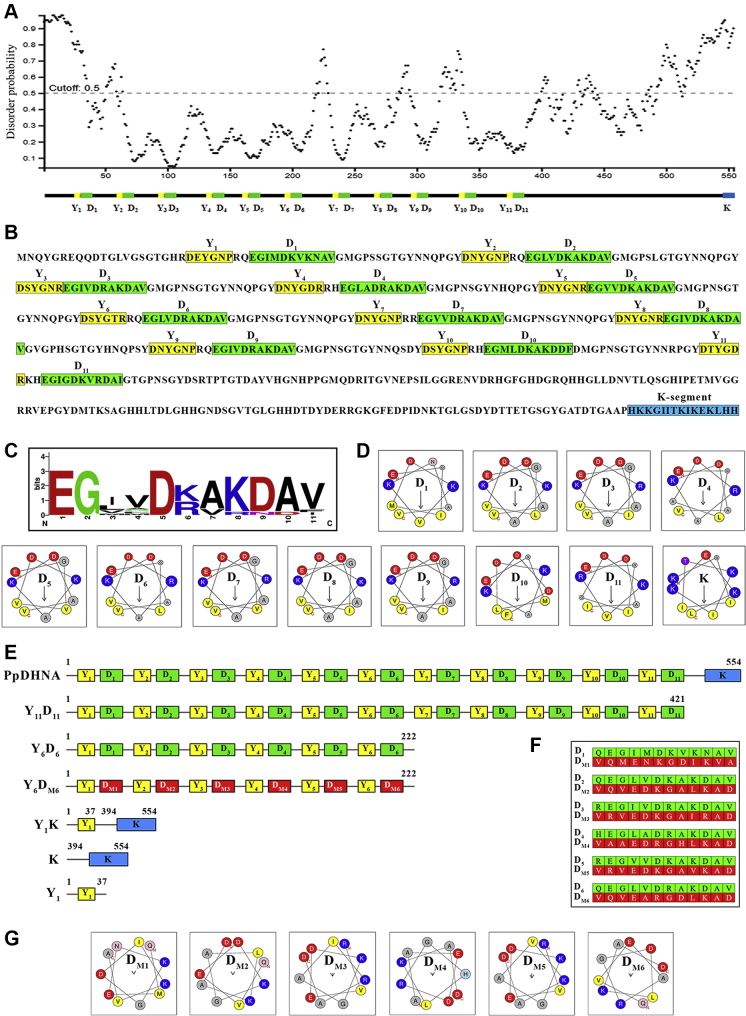
***In silico* structural analysis of PpDHNA and generation of deletion mutant proteins with altered secondary structure.***A*, DISOPRED analysis to predict dynamically disorder regions of PpDHNA. The *lower panel* shows the predicted position of Y-segments (*yellow boxes*) and D-segments (*green boxes*). *B*, sequence analysis of PpDHNA protein for the formation of probable helices. Y-segments are filled with *yellow boxes* and numbered as Y_1_-Y_11_, D-segments are marked with *green boxes* denoted with D_1_-D_11_, and K-segment is denoted with a *blue box*. *C*, LOGO representation of D-segment generated using WebLogo. Amino acids are color coded by their group type: *blue* for positively charged (Lys, Arg); *red* for negatively charged (Asp, Glu); *black* for hydrophobic (Ala, Val, Met, Phe, Iso, Leu), *green* for polar (Gly), and *purple* for neutral (Asn). The heights of the amino acids correspond to their conservation at that position. *D*, helical wheel projection model for different D-segments and K-segments of PpDHNA drawn using HeliQuest program. The N-terminal and C-terminal ends of the amino acid sequence are marked with a *red* N and C, respectively. The *arrow* indicates the hydrophobic moment. Hydrophobic residues are colored *yellow*, basic residues *blue*, acidic residues *red*, and glycine *gray*. *E*, schematic representation showing the organization of Y-, D-, and K-segments of PpDHNA and its deletion mutants (Y_11_D_11_, Y_6_D_6_, Y_6_D_M6_, Y_1_K, K, and Y_1_). The amino acid position of the corresponding region is given at the *top*. *F*, table showing amino acid sequences D-segments (D_1_-D_6_) in the PpDHNA protein along with the corresponding shuffled D-segments (D_M1_-D_M6_) in the deletion mutant Y_6_D_M6_. D-segments were shuffled by considering 12 amino acids instead of 11 amino acids for optimum disruption of amphipathic α-helical nature. *G*, helical wheel projection model for shuffled D-segments (D_M1_-D_M6_) present in the Y_6_D_M6_ deletion mutant. PpDHNA, *Physcomitrella patens* dehydrin.

As shown in Figure 1, PpDHNA is a Y_11_K type of DHN with 11 Y-segments (shown in yellow) and 1 K-segment (shown in blue). Since the K- and the Y-segments are already known to form putative α-helical structures under suitable environment, it is expected that a protein disorder analysis along the sequence will indicate structural order at these points, despite PpDHNA being an IDP. Indeed, an analysis of structural disorder, using the DISOPRED3 server, showed sudden dips in disorder at sequence positions corresponding to the 11 Y-segments (Fig. 1*A*). Analysis of disorder using another server (Glob plot 2) confirmed this observation (Fig. S1). The latter method uses the slope of the cumulative disorder propensity to predict disordered (positive slope) as well as putative folded domains (negative slope).

Although a canonical Y-segment typically extends over a stretch of six amino acid residues, interestingly, our analyses predicted a much longer stretch to be “ordered” at the 11 positions where putative Y-segments were identified. On closer inspection, a recurring stretch of amino acids, termed as the D-segment, was found to appear a few residues after the appearance of every Y-segment. The D-segment was associated with the sequence motif EGφφD(R/K)AKDAφ, where φ represents a hydrophobic residue (Fig. 1*B*). A LOGO representation of the D-segments is shown in Figure 1*C*. The Y-, D-, and K-segments along with their amino acid position in the PpDHNA protein sequence are shown in Tables S1 and S2.

To investigate the reason behind the predicted order of the D-segments, all D-segments were analyzed for their propensity to form amphipathic α-helix using the program HeliQuest. All D-segments were found to form identical distribution patterns of amphipathic α-helical segments (Fig. 1*D*), where polar residues (E, D) occur on one side and nonpolar residues (I, V, L, F, M) occur on the other side. A similar analysis of the K-segment showed optimum amphipathic α-helical arrangement by a stretch of 11 amino acid residues (15 amino acid residues reported elsewhere). The calculated hydrophobicities and hydrophobic moments of the D-segments are enlisted in Table S2.

### Generation of deletion mutants of PpDHNA

We generated a series of deletion mutants (Y_11_D_11_, Y_6_D_6_, Y_1_K, K, and Y_1_) based on bioinformatics analysis to determine the role of different segments present in the PpDHNA protein as shown in Table S3 and schematically represented in Figure 1*E*. In addition, another mutant Y_6_D_M6_ was generated where the number of amino acids was the same as the Y_6_D_6_ deletion mutant. However, the amino acid sequences in the D-segments were shuffled in the Y_6_D_M6_ mutant, keeping the amino acid composition similar to Y_6_D_6_ (Fig. 1*F*). The shuffling of the amino acid sequence was done to disrupt the amphipathic α-helix associated with each D-segment (Fig. 1*G*). Loss of hydrophobic moment and hydrophobicity upon shuffling of the D-segments are shown in Table S4.

The amplified PCR products of approximately 111 bp for Y_1_, 486 bp for K, 594 bp for Y_1_K, 666 bp for Y_6_D_6_, and 1263 bp for Y_11_D_11_ (shown in Fig. S2) were cloned and bacterially expressed for their corresponding proteins. The deletion mutants (Y_11_D_11_, Y_6_D_6_, Y_6_D_M6_, Y_1_K, K, and Y_1_) generated proteins of different sizes 50, 29, 29, 26, 43, and 30 kDa, respectively, as shown in Fig. S3, *A–D*. The deletion mutants (Y_11_D_11_, Y_6_D_6_, Y_6_D_M6_, and Y_1_K) were His-tag fused, whereas the K and Y_1_ were GST tagged. This accounts for the increase in size of K and Y_1_ against their original sizes of 17 and 4 kDa, respectively. Purified proteins with GST tags (Y_1_ and K) were immunoblotted with the corresponding anti-GST antibody, whereas, the His-tagged proteins (Y_11_D_11_, Y_6_D_6_, Y_6_D_M6_, and Y_1_K) were immunoblotted with the corresponding anti-HIS antibody as shown in Fig. S3*E*. The PpDHNA protein was prepared as reported previously (33).

### Circular dichroism spectra showed strong correlation with predicted secondary structure

**Figure 2:**
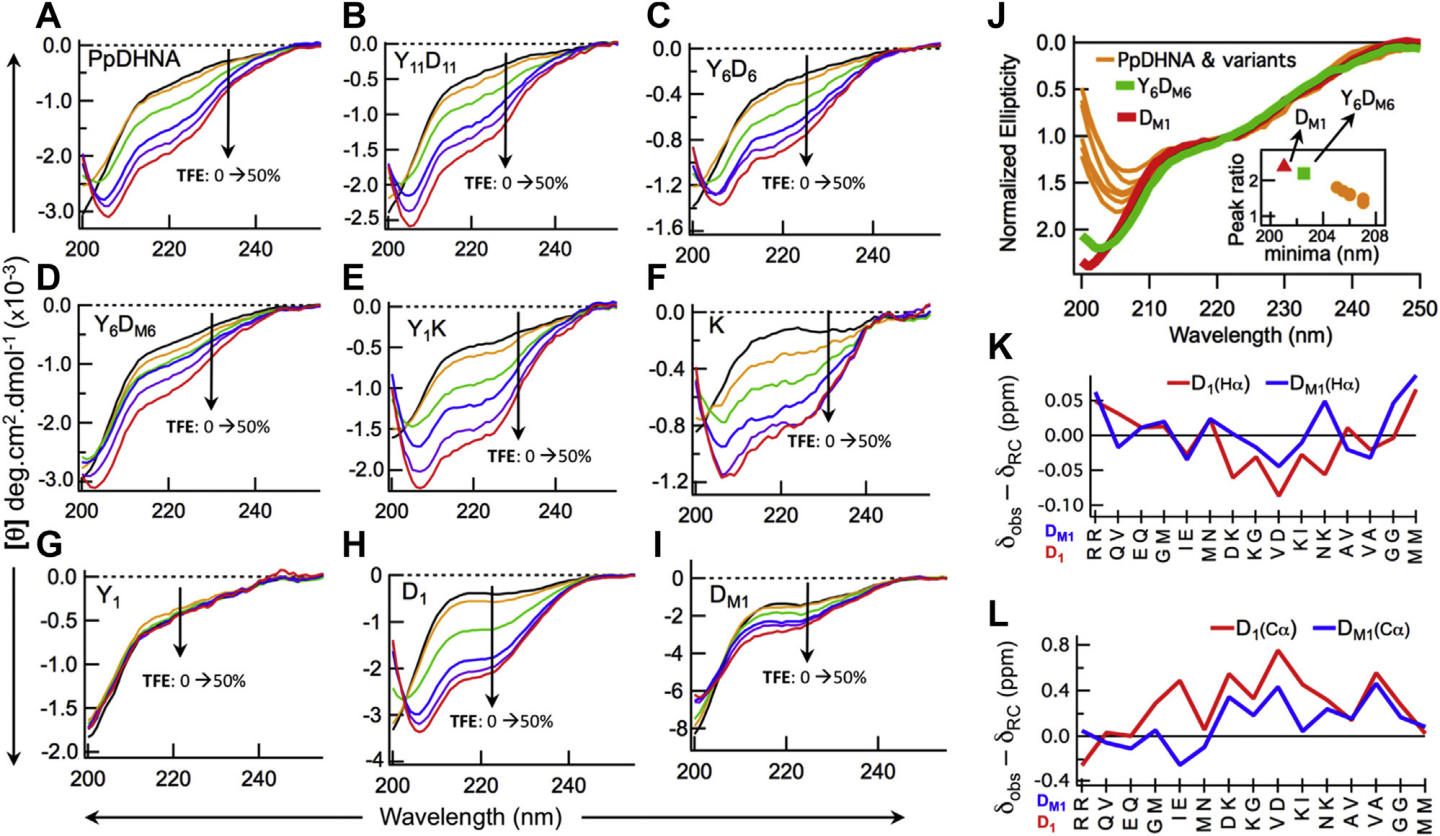
**CD spectra of PpDHNA and its deletion mutants (as a function of added trifluoroethanol) and NMR study of D-segment.***A*, PpDHNA, (*B*) Y_11_D_11_, (*C*) Y_6_D_6_, (*D*) Y_6_D_M6_, (*E*) Y_1_K, (*F*) K, (*G*) Y_1_, (*H*) D_1_, and (*I*) D_M1_. *J*, CD spectra of PpDHNA and its variants in the presence of 50% trifluoroethanol. The spectra have been normalized such that ellipticity at 222 nm is 1. The inset shows the variation of the peak ratio R (intensity of ππ∗_||_/intensity of nπ∗) *versus* the position of the ππ∗_||_ minimum. *K*, the difference between the observed and the random coil chemical shifts for ^1^Hα in peptides D_1_ and D_M1_. *L*, the difference between the observed and the random coil chemical shifts for ^13^Cα in peptides D_1_ and D_M1_. The chemical shift differences shown in (*K*) and (*L*) are average differences from three random coil values (36, 37, 38). PpDHNA, *Physcomitrella patens* dehydrin.

CD spectroscopic analysis was carried out to probe the secondary structure of the proteins. The purified proteins (PpDHNA and deletion mutants Y_11_D_11_, Y_6_D_6_, Y_6_D_M6_, Y_1_K, K, and Y_1_) were analyzed by CD. As shown in Figure 2, *A*–*G*, all protein variants (the uppermost black curve in each panel) were found to be disordered in an aqueous buffer. We analyzed the protein in presence of trifluoroethanol (TFE), a well-known helix-inducing solvent that is thought to mimic the membrane environment (18, 34). The CD spectra for all proteins in the presence of TFE (10%–50%) were also recorded, as shown in Figure 2, *A*–*G*. Except for Y_1_, in all cases, the CD spectra showed a gradual transition from disordered to α-helical conformation (characterized by double minima: one at ∼222 nm associated with the nπ∗ transition and another at ∼208 nm associated with the ππ∗_||_ transition). Despite the similarity in TFE-induced changes, helix induction for Y_6_D_M6_ was found to be distinctly different from the rest, especially when the ratio between the two minima (R = intensity of ππ∗_||_/intensity of nπ∗) was considered. The ratio R reflects the degree of helix formation (∼1 for 100% helix, > 1 otherwise) (35). The CD spectra of all proteins (at 50% TFE) are compared in Figure 2*J*, where the 222-nm ellipticities (nπ∗ transition) are normalized so that the relative intensities of the lower wavelength π-π∗_||_ transition reflect the value of R. Clearly, the π-π∗_||_ transition for Y_6_D_M6_ is more intense than for others. Also, as shown in the inset of Figure 2*J*, Y_6_D_M6_ stands out from the others, both in terms of the peak ratio R and the position of the π-π∗_||_ peak (∼208 nm for 100% helicity and <208 otherwise). The loss of TFE-induced helix induction upon shuffling of the D-segment indicates the inherent ability of the D-segment to adopt a helical conformation under a suitable environment. To further confirm this, CD spectra for both the isolated D-segment (D_1_) and the corresponding shuffled version (D_M1_) were recorded as a function of added TFE (Fig. 2, *H* and *I*). Both peptides were found to be disordered in the absence of TFE. Although TFE induced helicity in both, 50% TFE induced less helicity in D_M1_ compared with D_1_ (Fig. 2*J*).

### NMR chemical shifts indicated better helix-forming ability of D_1_ compared with D_M1_

Unlike CD spectroscopy, which reports the overall conformation of a polypeptide chain, NMR can provide residue-wise conformational bias in a peptide. Specifically, the chemical shifts δ (for both ^1^H^α^ and ^13^C^α^) of amino acid residues can indicate such biases. A residue is considered to be biased toward α-helix if {δ_H_α(obs) - δ_H_α(random coil)} < 0 and {δ_C_α(obs) - δ_C_α(random coil)} > 0. We have performed NMR experiments on both D_1_ and D_M1_ and have obtained the chemical shift values of H^α^ and C^α^ of all residues. The differences between the observed and the random coil chemical shifts (36, 37, 38) were plotted along the sequence for both the peptides. As shown in Figure 2, *K* and *L*, chemical shift differences (negative for ^1^H^α^ and positive for ^13^C^α^) indicated that the central part of D_1_ (DKVKN) is associated with a clear bias to form incipient α-helix in aqueous buffer. The shuffled helix D_M1_ also showed a slight tendency to form incipient α-helix in the aqueous buffer but consistently significantly less than D_1_ (throughout the sequence). In other words, NMR experiments confirmed the differential helix forming abilities of D_1_ and D_M1_ in the absence of TFE.

### Restoration of LDH activity under stress by dehydrins showed a positive correlation with the number of D-segments present

**Figure 3:**
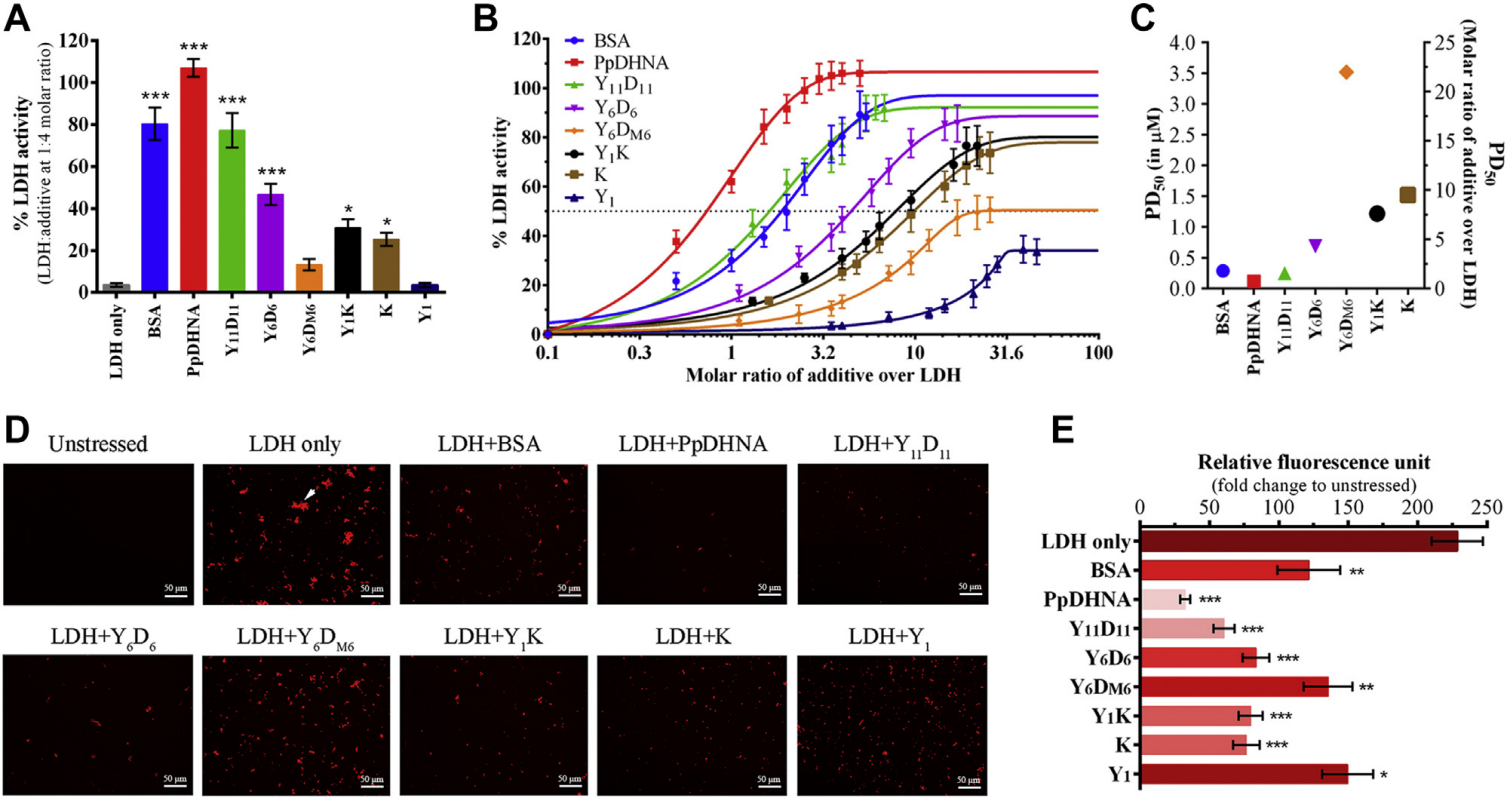
**Lactate dehydrogenase (LDH) protection activity of PpDHNA and its deletion mutants under high-temperature stress conditions.***A*, LDH activity after high-temperature stress treatment at 54 °C for 10 min in the absence (LDH only) or presence of PpDHNA and its deletion mutants (at 1:4 molar ratio). *B*, LDH activity after high-temperature stress with increasing molar ratio (from 1:0.5 to 1:40 molar ratio) of LDH: PpDHNA and its deletion mutant; data were fit to a nonlinear regression (sigmoidal) curve. *C*, comparison of PD_50_ value of PpDHNA and its deletion mutant showing the relationship between molar ratio and required molar concentration simultaneously. *D*, fluorescence microscopic images of LDH aggregates visualized by Congo red staining after high-temperature stress in the presence and absence of PpDHNA and its deletion mutants under 20× objective; Scale bars, 50 μm. *E*, spectrofluorometric quantification of LDH aggregates at excitation and emission at 496 and 614 nm, respectively, and represented in terms of the fold change of relative fluorescence unit with respect to unstressed LDH enzyme. Bovine serum albumin served as a positive control for all the experiments. Histogram bars have been marked with a color intensity gradient (higher value is represented by *higher color intensity*). Data are represented as means ± SD (n = 3). ∗*p* < 0.01, ∗∗*p* < 0.001 and ∗∗∗*p* < 0.0001 show statistically significant differences with control, *i.e.*, LDH. PpDHNA, *Physcomitrella patens* dehydrin.

Upon high-temperature treatment at 54 °C for 10 min, LDH lost most of its activity, retaining only about 5% of its initial activity (100%). However, the presence of a minimal concentration of ∼1250 nM of PpDHNA (1:4 molar ratio of LDH:additive protein) could restore its original activity. In comparison, a 1:4 molar ratio of all other deletion mutants (Y_11_D_11_, Y_6_D_6_, Y_6_D_M6_, Y_1_K, K, and Y_1_) showed a variable degree of protection (as shown in Fig. 3*A*). The protective activity of the additive proteins showed a positive correlation with the number of D-segments (amphipathic α-helix) and also the size of the proteins. The protective activity increased as the molar ratios of the additive proteins were increased (Fig. 3*B*). The relative activity of LDH, when incubated with PpDHNA, showed maximum protective activity (∼100%) compared with Y_11_D_11_ (77%) and Y_6_D_6_ (48%). This difference in the level of protection reflects the importance of the number of D-segments as well as the size of the protein for protection. Of interest, Y_11_D_11_ showed protection similar to a known protectant bovine serum albumin (BSA) (80%). The proteins with K-segment (Y_1_K and K) could retain about 25% to 30% of LDH activity (Fig. 3*A*) under stress. Of note, upon increasing the molar concentration (1:20), the maximum protection provided by Y_1_K and K was about 75% (Fig. 3*B*). However, Y_6_D_M6_, with disrupted amphipathic α-helices, could only restore 13% of LDH activity (Fig. 3*A*). Even on increasing the molar ratio to 1:22, the maximum restoration was 50% only (Fig. 3*B*). Therefore, the effect of Y_6_D_M6_ remained as an outlier when compared with other deletion mutants, in terms of the PD_50_ value (shown in Fig. 3*C*). The distribution of PD_50_ values of PpDHNA and its mutant proteins correlates well with their capacity of amphipathic α-helix formation as well as the size of the proteins. Finally, Y_1_ provides the least amount of protection, reaching a maximum of 36% at 1:40 molar ratio. Therefore, no PD_50_ value could be assigned to it (Fig. 3*B*). Our experiments are in close agreement with the previous reports showing that the K-segment (amphipathic α-helix), as well as the size of the protein, has a definite role in LDH protection (39). Furthermore, it was also proved beyond doubt that, apart from the K-segment, other amphipathic α-helices (like in the D-segments) were also capable of showing the protective activity.

### D-segments have the potential to inhibit LDH aggregation as reflected by PpDHNA and its deletion mutants

LDH loses its activity at high-temperature forming aggregates, which could be visualized by staining with Congo red (40). LDH was found to lose its maximum activity (∼95%) at 54 °C when incubated for 10 min. LDH showed substantial aggregation as compared with the control (Fig. 3*D*). The results obtained were also quantified fluorometrically (Fig. 3*E*). However, in presence of PpDHNA, the aggregate formation was significantly less. Similarly, deletion mutants with higher number of D-segments (Y_11_D_11_ and Y_6_D_6_) showed very little LDH aggregation. Of note, aggregation was much less in the case of Y_11_D_11_ as compared with Y_6_D_6_. The Y_1_ mutant, lacking the D-segment, showed a greater amount of Congo red–stained aggregates. Moreover, Y_6_D_M6_ with shuffled amino acid sequence in the D-segments also was associated with higher aggregation of LDH as compared with Y_6_D_6_. Therefore, it can be conclusively stated that the arrangement of amino acids present in the D-segment is particularly important. Y_1_K and K-deletion mutants were also associated with some degree of protein aggregation, which was slightly more in the case of the K-deletion mutant. Since the K-segment also forms an amphipathic α-helix, the presence of this segment is important for arresting aggregation. Considering the size of the proteins used in this assay, a positive correlation exists between the size and the protective activity. This confirms the number of D-segments and the size of the protein as two important features determining the protective activity of PpDHNA.

### PpDHNA provides stress tolerance to *E. coli* cells through proteome protection mediated by D-segments

**Figure 4:**
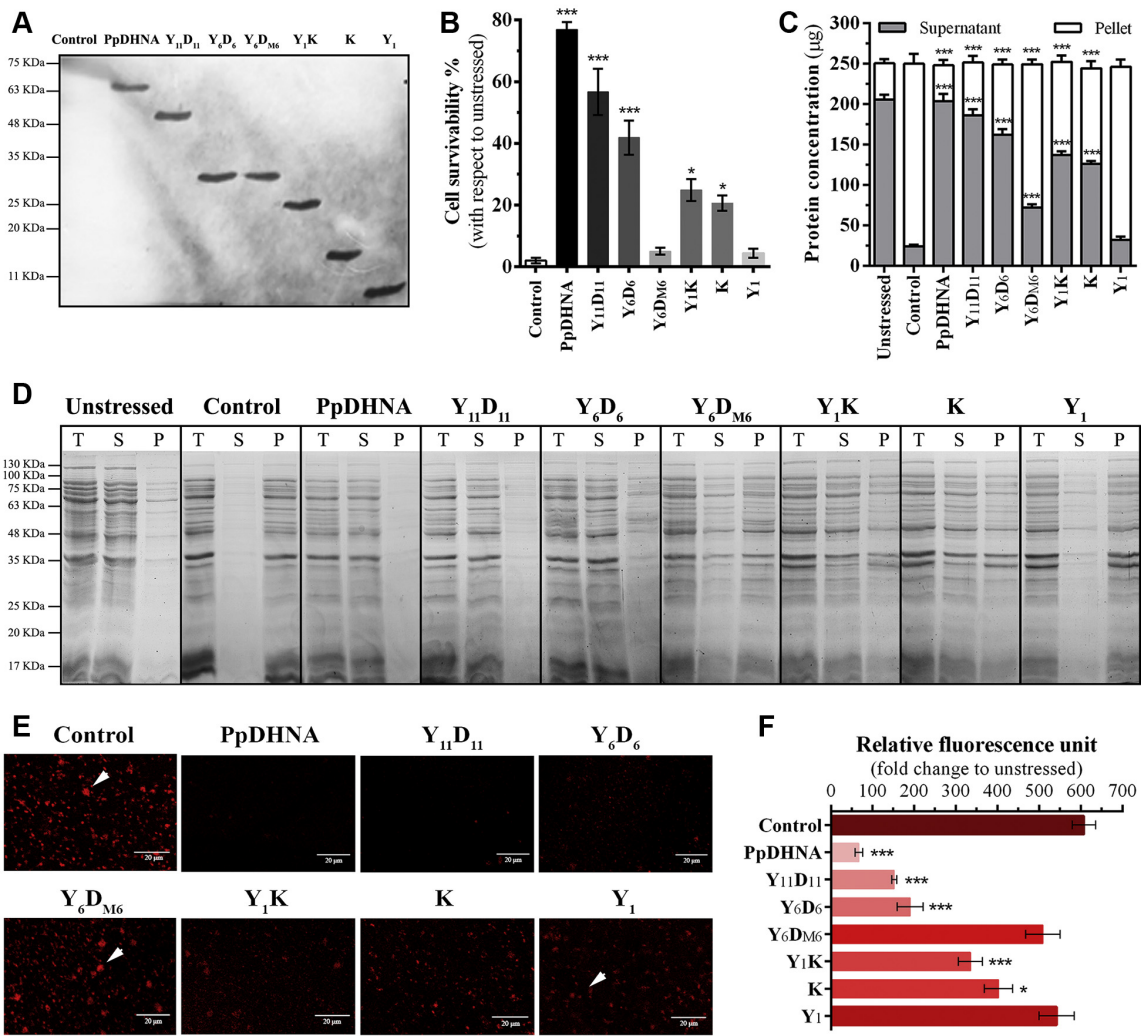
**Cell survivability and *in vivo* proteome protection assay of *E. coli* cells expressing PpDHNA and its deletion mutants upon high-temperature stress.** Cells were induced with 1 mM of IPTG for 20 min and exposed to high-temperature stress at 54 °C for another 20 min. *A*, immunoblot detection of recombinant proteins in *E. coli* cells using anti-HIS antibody. *B*, percentage of cell survivability of *E. coli* cells overexpressing the respective proteins; calculated with respect to the unstressed nontransformed *E. coli* cell. *C*, quantification of protein content present in supernatant and pellet fraction of unstressed and stress-treated *E. coli* cells. *D*, analysis of unstressed and stress-treated *E. coli* cells on 12% SDS-PAGE where *(T)* represents total cellular protein, *(S)* protein present in the supernatant fraction, and *(P)* protein present in the pellet fraction. *E*, confocal images of *E. coli* cells under 40× objective upon staining with aggregate marker dye ProteoStat; aggregates are marked with *white arrowhead*; scale bars, 20 μm. *F*, spectrofluorometric quantification fluorescence level for intercellular aggregates at excitation and emission of 550 and 603 nm, respectively, and represented in terms of the fold change of relative fluorescence unit with respect to unstressed cells. Nontransformed *E. coli* Rosetta (DE3) pLysS bacterial host strain exposed to stress was used as a control for all the experiments. Histogram bars have been marked with a color intensity gradient (higher value represents *higher color intensity*). Data are represented as means ± SD (n = 3). ∗*p* < 0.01, and ∗∗∗*p* < 0.0001 show statistically significant differences with control. PpDHNA, *Physcomitrella patens* dehydrin.

PpDHNA and its deletion mutants overexpressed in *E. coli* cells exhibited a different degree of protection against high-temperature (54 °C). The recombinant proteins were expressed equally as shown in immunoblot analysis (Fig. 4*A*). Both Y_6_D_M6_ and Y_1_ showed almost negligible protection upon stress treatment, *i.e.*, 5% and 4% cell survivability similar to nontransformed (NT) cells (control) (Fig. 4*B*). However, *E. coli* cells overexpressing D-segment bearing proteins PpDHNA, Y_11_D_11_, and Y_6_D_6_ showed better cell viability of 77%, 57%, and 42%, respectively. On the other hand, Y_1_K and K recombinant proteins, containing single K-segments, were able to protect 25% and 21% *E. coli* cells, respectively.

The protective effect of the overexpressed recombinant proteins was evaluated by subjecting the *E. coli* cells to high-temperature stress. The protein extract was quantified and analyzed on 12% SDS-PAGE (Fig. 4, *C* and *D*). NT *E. coli* cells were used as a control in these experiments. The control cells showed a higher amount of protein in the pellet fraction as compared with the supernatant. However, *E. coli* cells overexpressing PpDHNA protein showed a higher amount of protein in the supernatant (211 μg) (Fig. 4*C*). Similarly, Y_11_D_11_ and Y_6_D_6_ also showed comparable amounts of proteins (186 and 162 μg, respectively) in the supernatant. Cells expressing Y_1_K and K were able to protect their proteomes to a lesser degree. A considerable amount of the protein (115 and 118 μg for Y_1_K and K, respectively) was observed in the pellet fraction (Fig. 4, *C* and *D*). However, for Y_6_D_M6_ and Y_1_, a minimal amount of protein (72 and 32 μg, respectively) was found in the supernatant fraction. This points toward the fact that the overexpressed proteins Y_6_D_M6_ and Y_1_ were insufficient for rendering protection (Fig. 4, *C* and *D*). The presence of the proteins in the soluble fraction under temperature stress conditions correlated well with the cell survivability dataset. These data highlight the importance of the D-segment in imparting high-temperature tolerance through proteome protection.

### Inhibition of intracellular protein aggregation in *E. coli* cells as a function of D-segment

*In vivo* protein aggregation was evaluated under high-temperature stress on *E. coli* cells transformed with PpDHNA and its deletion mutants. ProteoStat staining revealed that the D-segment bearing proteins PpDHNA, Y_11_D_11_, and Y_6_D_6_ significantly lowered the extent of aggregate formation as shown in Figure 4*E* and quantitatively represented in Figure 4*F*. In contrast, Y_6_D_M6_ and Y_1_ showed a higher amount of intracellular protein aggregation as indicated by higher fluorescence (Fig. 4, *E* and *F*). Therefore, these data are in close agreement with the proteome protection as well as cell survivability assay. Therefore, D-segments are crucial for high-temperature tolerance of *E. coli* cells.

### D-segments enhanced the stress tolerance in transformed tobacco plants

To confirm the involvement of the D-segment in the protective activity of PpDHNA and its deletion mutants, further functional characterization was carried out by overexpressing them in tobacco plants. The almost similar expression levels of GFP in qRT-PCR and immunoblot analysis of transformed lines of PpDHNA and its deletion mutants (Y_11_D_11_, Y_6_D_6_, Y_6_D_M6_, Y_1_K, Y_1_, and K) indicated that all deletion mutant transformed plants had similar copy number (shown in Fig. S4, *A* and *B*).

**Figure 5:**
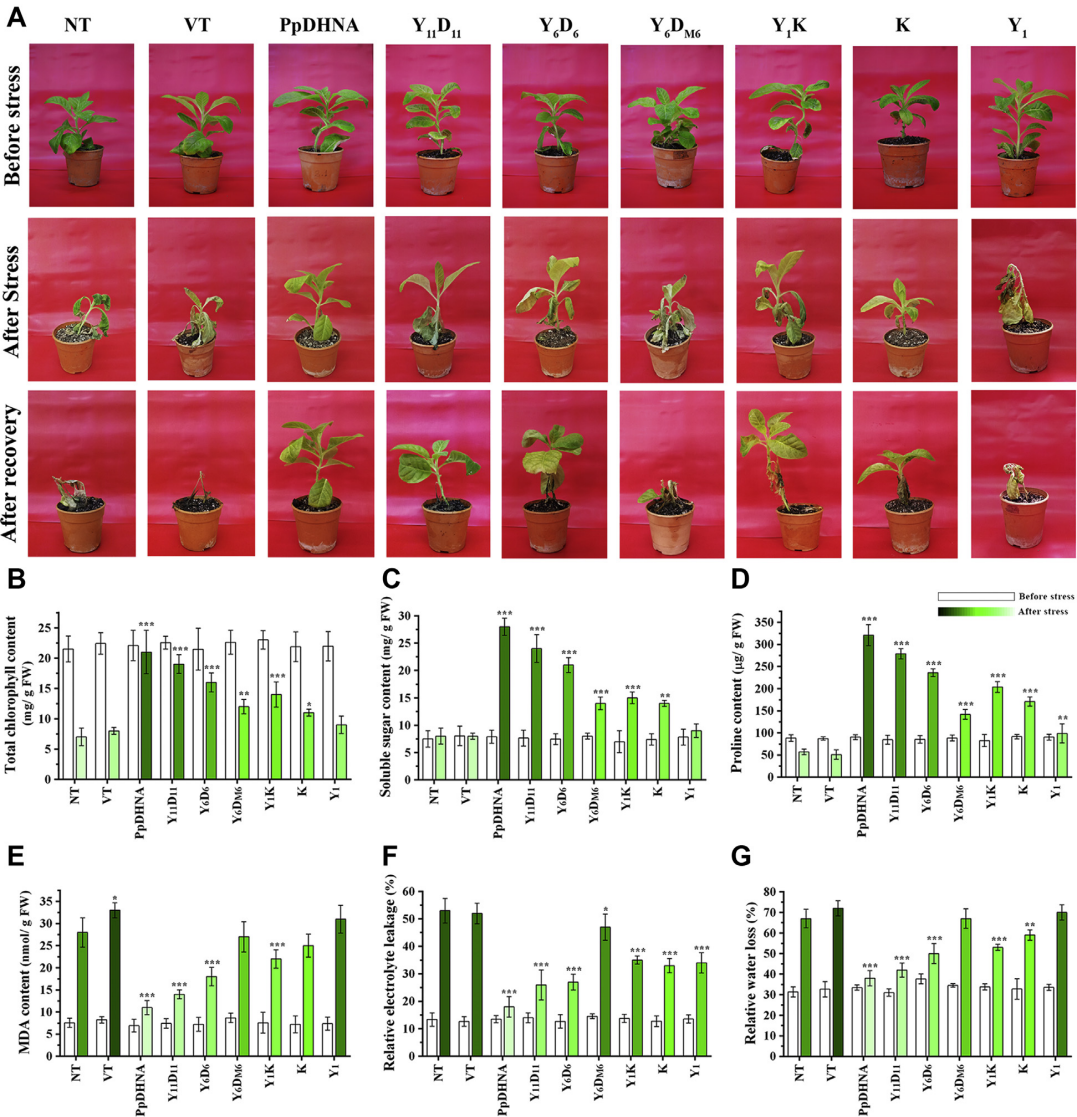
***In planta* stress tolerance assay of transformed *Nicotiana tabacum* plants harboring PpDHNA and its deletion mutants**. *A*, comparative representative figure of transformed plants (PpDHNA, Y_11_D_11_, Y_6_D_6_, Y_6_D_M6_, Y_1_K, K, and Y_1_) along with nontransformed (NT) and vector transformed (VT) plants before and after 14 days treatment of high-temperature coupled with dehydration stress according to the stress regime. The *lower panel* shows the stress-treated plants after 10 days of recovery at normal growth conditions (24 °C, 16 h light/8 h dark). Assays were performed with three randomly selected lines of each transformed protein. The stress tolerance ability of each overexpressed protein was represented. Analysis of PpDHNA and its deletion mutants transformed plants along with NT, VT plants for different stress treatment: *B*, total chlorophyll content, (*C*) soluble sugar content, (*D*) proline content, (*E*) MDA content, (*F*) relative electrolytic leakage, and (*G*) relative water loss. Unstressed transformed plants were used as a control for each experiment. Histogram bars for after stress have been marked with color intensity gradient (higher value represents *higher color intensity*) and noncolor (*white*) for before stress bars. Data are represented as means ± SD (n = 9). ∗*p* < 0.01, ∗∗*p* < 0.001, and ∗∗∗*p* < 0.0001 show statistically significant differences with control, *i.e.*, NT. PpDHNA, *Physcomitrella patens* dehydrin.

High-temperature coupled with desiccation stress treatment showed significant differences in the phenotypic condition of the transformed lines. The data showed a positive correlation with the number of amphipathic α-helices carried by the overexpressed plants (Figs. 5*A* and S5). Upon stress treatment, PpDHNA, Y_11_D_11_, and Y_6_D_6_ showed lesser wilting conditions and recovered quickly after the stress was removed. On the other hand, Y_6_D_M6_ and Y_1_ failed to protect the plants from the applied stress conditions. The plants showed severe wilting, and the recovery rate was slower. The K-segment bearing deletions Y_1_K and K displayed better tolerance with a moderate recovery rate.

### Measured physiological parameters reflected better tolerance in transformed tobacco plants with more D-segments under stress

PpDHNA and its deletion mutants overexpressed in transformed tobacco plants were studied for different biochemical parameters, with and without stress. Upon stress, the chlorophyll content (Fig. 5*B*) of the transformed plants decreased in the order PpDHNA, Y_11_D_11_, Y_6_D_6_ and the rest (Y_6_D_M6_, Y_1_K, K, and Y_1_) showed similar values. Thus, the first three coped with stress the best. This result correlated with the soluble sugar and proline content, both indicators of how good the plant is coping with stress. Upon stress, the sugar and the proline content (Fig. 5, *C* and *D*) of the transformed plants also decreased in an order similar to that of the chlorophyll content. Together, they point toward the superiority of PpDHNA, Y_11_D_11_, Y_6_D_6_ variants in coping with stress. The four poorly performing variants (Y_6_D_M6_, Y_1_K, K, and Y_1_) also showed the highest values of malondialdehyde (MDA) (Fig. 5*E*), relative electrolyte leakage (Fig. 5*F*), and relative water loss (Fig. 5*G*). Overall, a decrease in the number of D-segments decreased chlorophyll content, soluble sugar, and proline content. The D-segment containing variants (PpDHNA, Y_11_D_11_, and Y_6_D_6_) also maintained lower electrolyte loss, MDA content, and higher water-retention capacity. Of interest, variants containing the K-segment (Y_1_K and K) were able to mitigate stress moderately when compared with the NT.

### D-segments inhibit stress-mediated *in planta* protein aggregation in transformed tobacco plants

**Figure 6:**
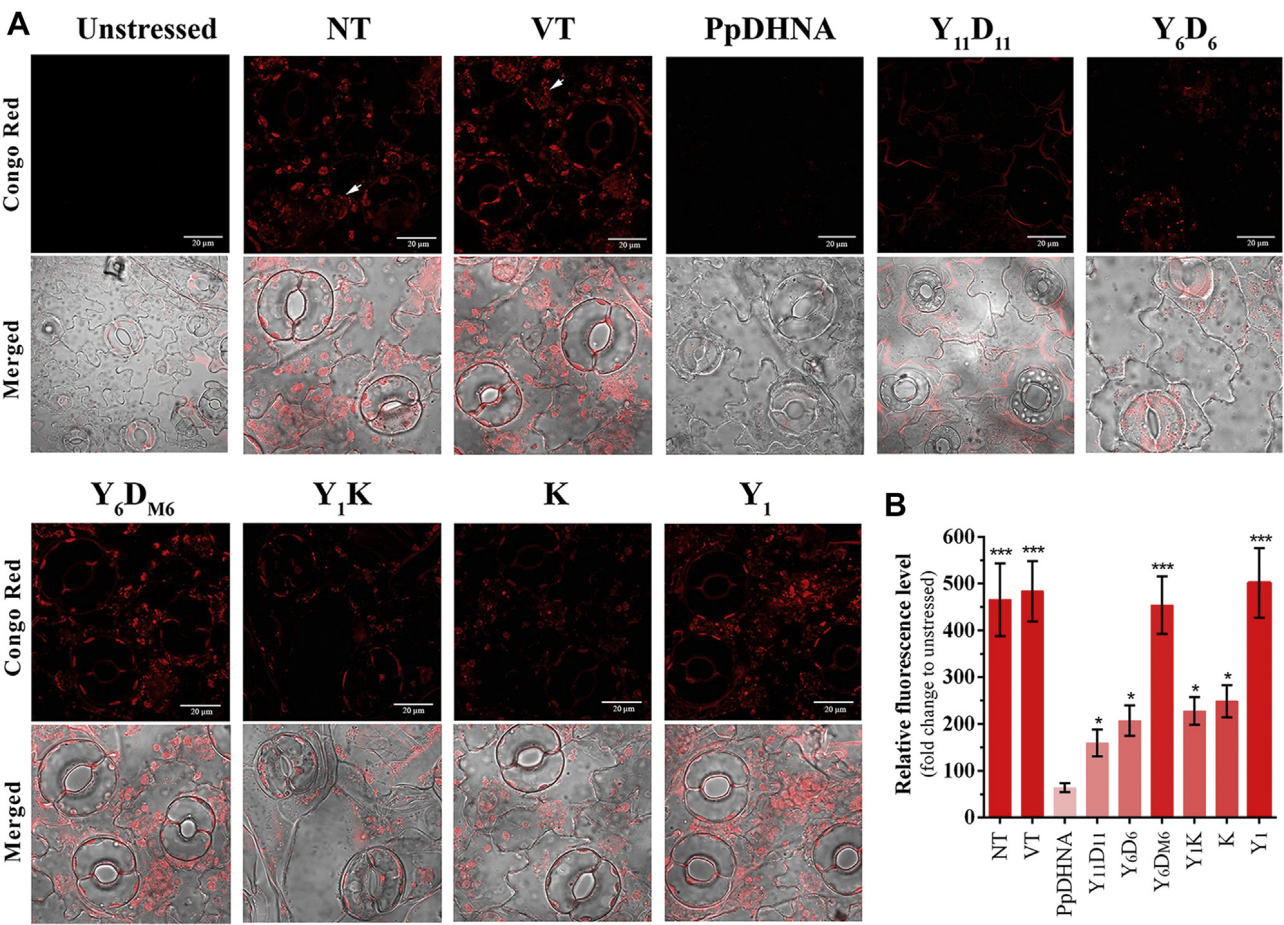
***In planta* protein aggregation protection assay**. *A*, confocal microscopic images of leaf peels stained with Congo red from PpDHNA and its deletion mutants transformed plants along with nontransformed (NT) and vector transformed (VT) plants before and after subjecting to 4 h of high-temperature stress treatment at 48 °C. Stress-induced subcellular protein aggregates were marked with *white arrowheads*. *B*, fluorescence intensity of the protein aggregates quantitatively estimated from the microscopic images of each transformed plants using ImageJ software. Data have been shown in terms of fold change to the unstressed set, wherein mean gray value was used for the analysis. Data are represented as means ± SD (n = 3 microscopic field). ∗*p* < 0.01, and ∗∗∗*p* < 0.0001 show statistically significant differences with unstressed plants. PpDHNA, *Physcomitrella patens* dehydrin.

An *in planta* protein aggregation study was carried out in transformed plants overexpressing PpDHNA and its deletion mutants. Upon high-temperature stress, a significantly lower amount of intracellular protein aggregation was observed in leaf peel sections of PpDHNA and Y_11_D_11_ transformed plants. The NT and vector transformed (VT) plants showed the presence of large and highly fluorescent protein aggregates in the cytoplasm (Fig. 6, *A* and *B*). Of interest, a clear difference between Y_6_D_6_ and Y_6_D_M6_ validated the need for amphipathic α-helices in preventing cellular protein aggregation. Similarly, Y_1_ transformed plants also failed to protect the cellular proteins from aggregating, alike NT and VT plants. Moreover, transformed plants overexpressing Y_1_K and K mutants showed fewer aggregates. The antiaggregation ability was relatively higher for Y_6_D_M6_ and Y_1_ transformed plants as shown by their mean fluorescence level (Fig. 6*B*).

Furthermore, these intracellular aggregates were isolated from stress-treated plants. A large number of aggregates were found to be formed in Y_1_ and Y_6_D_M6_ transformed plants. There was almost no aggregate formation in PpDHNA, Y_11_D_11_, and Y_6_D_6_ plants (Fig. S6). However, transformed plants overexpressing Y_1_K and K deletion mutants showed fewer aggregates (Fig. S6). Hence, the data further confirmed the role of D-segments in protecting the cells from high-temperature stress-mediated protein aggregation.

### Bimolecular fluorescence complementation analysis demonstrates the ability of amphipathic α-helices for *in planta* DHN–DHN interactions

**Figure 7:**
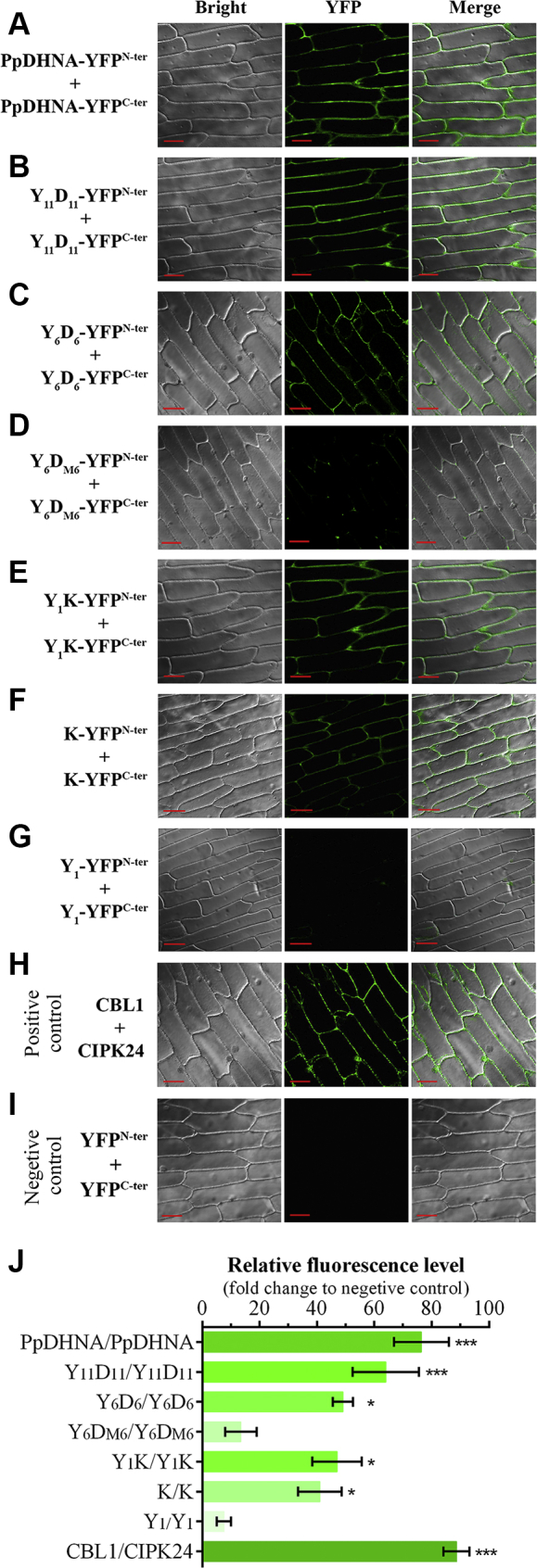
**Interaction analysis of PpDHNA and its deletion mutants (Y**_**11**_**D**_**11**_**, Y**_**6**_**D**_**6**_**, Y**_**6**_**D**_**M6**_**, Y**_**1**_**K, K, and Y**_**1**_**) by bimolecular fluorescence complementation analysis.** PpDHNA and its deletion mutants were individually cloned in both pCAMBIA1301-YFP^N-ter^ and pCAMBIA1301-YFP^C-ter^ binary vectors. Protein–protein interaction was studied by cotransformation of the tagged YFP^N-ter^ and YFP^C-ter^ proteins in onion epidermal cells by *Agrobacterium*-mediated transformation. Epidermal peels were analyzed under confocal microscope using YFP filter and photographed with 20× objective. *A*, PpDHNA-YFP^N-ter^/PpDHNA-YFP^C-ter^, (*B*) Y_11_D_11_-YFP^N-ter^/Y_11_D_11_-YFP^C-ter^, (*C*) Y_6_D_6_-YFP^N-ter^/Y_6_D_6_-YFP^C-ter^, (*D*) Y_6_D_M6_-YFP^N-ter^/Y_6_D_M6_-YFP^C-ter^, (*E*) Y_1_K-YFP^N-ter^/Y_1_K-YFP^C-ter^, (*F*) K-YFP^N-ter^/K-YFP^C-ter^, (*G*) Y_1_-YFP^N-ter^/Y_1_-YFP^C-ter^, (*H*) CBL1 and CIPK24, and (*I*) YFP^N-ter^/YFP^C-ter^. *Red scale bars* represent 50 μm. *J*, YFP fluorescence intensity of interacting proteins was quantitatively estimated from the microscopic images of each cotransformed onion epidermal cells using ImageJ software. Data have been shown in terms of fold change to the negative control set, wherein mean gray value was used for the analysis. Data are represented as means ± SD (n = 3 microscopic field). ∗*p* < 0.01, and ∗∗∗*p* < 0.0001 show statistically significant differences with negative control.

To investigate whether the DHN molecule can self-interact and to understand the role of amphipathic α-helices for the same, a bimolecular fluorescence complementation (BiFC) assay was performed. The analysis showed a strong YFP signal, localized at the plasma membrane transiently coexpressing PpDHNA/PpDHNA and Y_11_D_11_/Y_11_D_11_ (Fig. 7, *A*, *B*, and *J*). A relatively lower fluorescence signal was also obtained confined to the plasma membrane region for the combination of Y_6_D_6_/Y_6_D_6_ (Fig. 7, *C* and *J*). Of interest, the Y_6_D_M6_/Y_6_D_M6_ combination failed to constitute the YFP fluorophore, exhibiting almost no fluorescence signal (Fig. 7, *D* and *J*). Furthermore, a lower fluorescence signal was obtained for Y_1_K/Y_1_K and also for K/K in the cellular membrane as well as inside the nuclei (Fig. 7, *E* and *F*). No fluorescence was detected in the case of Y_1_/Y_1_ combination (Fig. 7*G*). CBL1 and its membrane interacting partner CIPK24 served as the positive control (Fig. 7*H*), whereas the pCAMBIA1301-YFP^N-ter^ and YFP^C-ter^ vector combination served as the negative control (Fig. 7*I*). These results indicated that PpDHNA could self-associate with amphipathic α-helices, whether its K- or D-segment, being the major contributor behind such interactions. The absence of such self-association property for Y_6_D_M6_ and Y_1_, along with a moderate degree of interaction in K and Y_1_K, emphasizes the role of amphipathic α-helices for DHN–DHN interaction in PpDHNA.

### The wildtype amphipathic D-segments self-associate but not the shuffled variants

**Figure 8:**
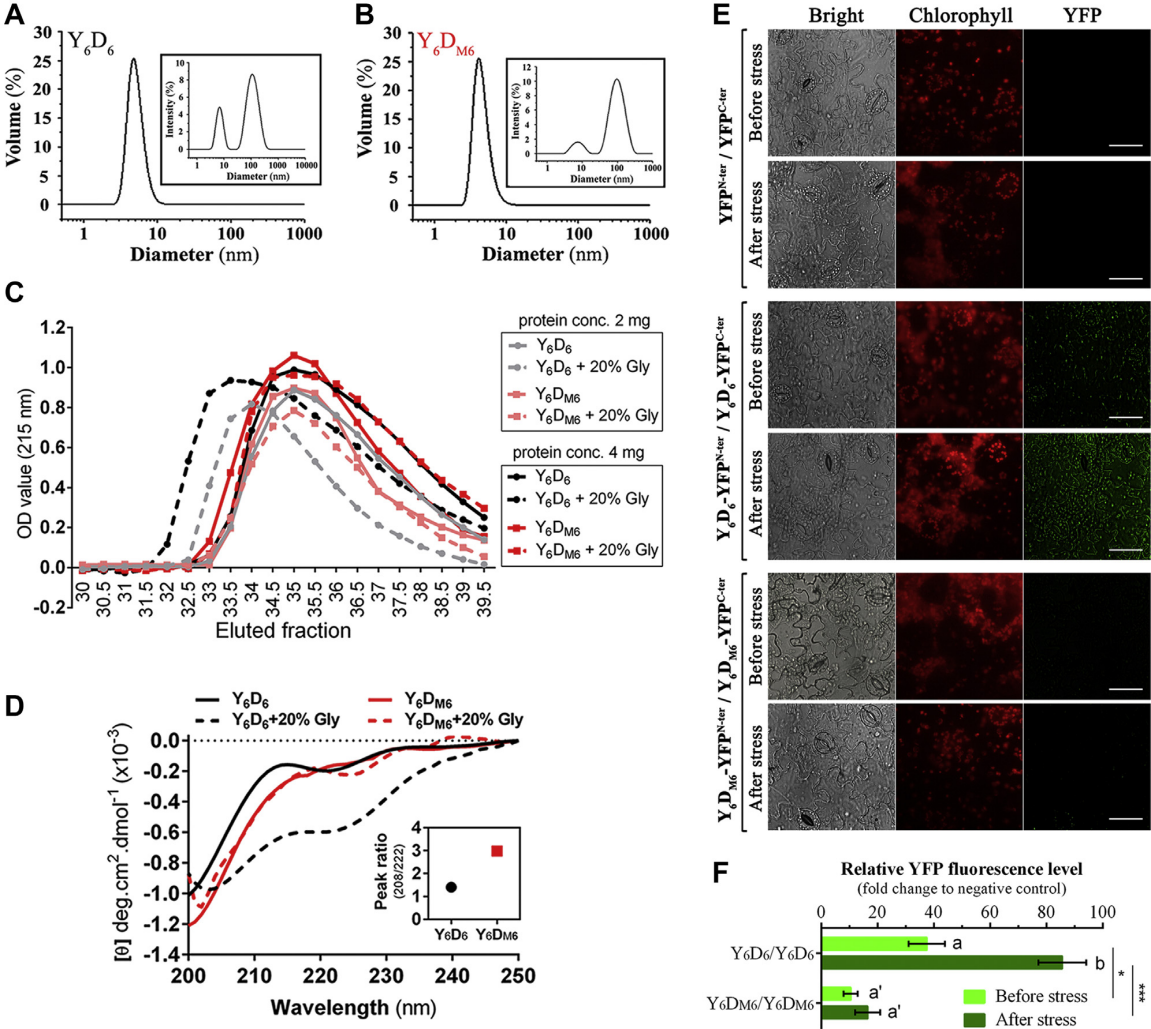
**Self-association property determination of Y**_**6**_**D**_**6**_**and Y**_**6**_**D**_**M6**_**.** Dynamic light scattering spectra of (*A*) Y_6_D_6_ and (*B*) Y_6_D_M6_ showing scattering volume *versus* size distribution histogram of proteins. Scattering intensity *versus* size distribution histogram spectra were represented in the inset of the corresponding proteins. *C*, size exclusion chromatographic profile of Y_6_D_6_ and Y_6_D_M6_ proteins at increasing protein concentration 2 and 4 mg in the presence and absence of 20% glycerol. *D*, CD spectra of Y_6_D_6_ and Y_6_D_M6_ in presence of 20% glycerol (Gly). The inset shows the variation of the peak ratio R (208/222 nm). *E*, fluorescence microscopic images of *N. tabacum* leaf peels showing the YFP fluorescence upon high-temperature stress and unstressed condition through bimolecular fluorescence complementation analysis. *N. tabacum* leaves were transiently transformed with Y_6_D_6_-YFP^N-ter^/Y_6_D_6_-YFP^C-ter^ and Y_6_D_M6_-YFP^N-ter^/Y_6_D_M6_-YFP^C-ter^ constructs, before stress treatment. YFP^N-ter^/YFP^C-ter^ was used as a negative control for the experiments. *White scale bars* represent 50 μm. *F*, YFP fluorescence intensity of interacting proteins was quantitatively estimated from the microscopic images of each cotransformed tobacco leaf peels using ImageJ software. Data have been shown in terms of fold change to the negative control set, wherein mean gray value was used for the analysis. Data are represented as means ± SD (n = 3 microscopic field). ∗*p* < 0.01 and ∗∗∗*p* < 0.0001 show statistically significant differences between the individual constructs, whereas similar letters represent no significant differences within each construct when compared between before and after stress conditions.

Two proteins (Y_6_D_6_ and Y_6_D_M6_) were chosen for self-association analysis, as both these proteins have the same number of amino acid residues and composition. Dynamic light scattering experiment revealed almost identical size distribution histogram spectra for Y_6_D_6_ and Y_6_D_M6_ protein (Fig. 8, *A* and *B*). Therefore, the average diameter obtained from the size distribution pattern of Y_6_D_6_ and Y_6_D_M6_ molecules was the same, *i.e.*, *d*_*H*_≅ 4.5 nm.

The same was proved in the gel filtration experiment, as Y_6_D_6_ and Y_6_D_M6_ eluted in the same fraction when aqueous buffer was used as the mobile phase (Fig. 8*C*). In addition, this phenomenon was found to be consistent with increasing protein concentrations. However, in presence of 20% glycerol (helix-inducing agent), Y_6_D_6_ eluted in the earlier fractions, which shifted more upon increasing protein concentration, indicative of self-association. On the contrary, Y_6_D_M6_ continued to be eluted in the same fraction with 20% glycerol and even on increasing the protein concentration. Thus, the absence of any association between the molecules of Y_6_D_M6_ was convincingly proved (Fig. 8*C*).

To dissect out whether this self-association nature is associated with amphipathic α-helix (D-segment) transition, CD analyses of both the proteins were carried out in the presence and absence of 20% glycerol. Of interest, coil-to-helix transition was visualized for Y_6_D_6_; however, Y_6_D_M6_ showed no helix induction (Fig. 8*D*). The minima of 208/222 nm peak ratio (R) indicate the incipient α-helix formation ability of Y_6_D_6_ in the presence of 20% glycerol (inset of Fig. 8*D*). As both the proteins were of the same size and composition but with shuffled D-segments, this convincingly showed that the association of Y_6_D_6_ is linked with the D-segments. Hence, our experimental results indicate an association owing to the helix formation propensity of Y_6_D_6_.

### Self-interaction of DHN is required for stress protection

Stress-induced self-association of DHN was analyzed using Y_6_D_6_ and its shuffled variant Y_6_D_M6_. The BiFC dataset of tobacco leaf analysis revealed that, in case of Y_6_D_6_/Y_6_D_6_ constructs, high-temperature stress exposure led to significant amplification of YFP fluorescence in tobacco leaf peels as compared with unstressed plants (Fig. 8, *E* and *F*). Moreover, as an effect of stress exposure, the YFP fluorescence was observed throughout the cytoplasm and the membrane regions of tobacco epidermal cells. In contrast, the leaf peels of the Y_6_D_M6_/Y_6_D_M6_ construct did not show any changes in YFP fluorescence, even upon stress treatment. The negative control also showed similar results (Fig. 8, *E* and *F*). This observation clearly established the fact that the D-segment was essential for self-association or self-interaction and that self-interaction is a requisite for stress protection.

## Discussion

### A novel amphipathic α-helix forming D-segment in PpDHNA

DHNs are an important class of plant proteins whose function is to abate the effects of abiotic stress. The functional role of DHNs become even more crucial in model organisms like *Physcomitrella* that can tolerate extreme desiccation stress. *Physcomitrella* dehydrin PpDHNA has been shown to provide a protective function during osmotic and/or drought stress (30, 32, 33). However, the exact molecular mechanism of how it functions is unclear.

The current study was undertaken to look carefully at any idiosyncratic features in PpDHNA that might be responsible for its unusually high degree of stress protection. A major finding of the current study is the identification of a novel D-segment in PpDHNA that appears adjacent to the Y-segment. The D-segment, with the sequence motif EGφφD(R/K)AKDAφ, where φ is a hydrophobic residue, showed amphipathic α-helix gaining propensity, similar to the K-segment. Decades of research on DHNs have shown the K-segment to be one important factor behind DHN function (13, 15, 41, 42). The K-segment has been shown to adopt an amphipathic α-helix organization under water-deficit stress conditions (1).

To analyze the protective ability of the identified D-segment, along with the role of any other segments (Y and K), several deletion mutants were generated (Y_11_D_11_, Y_6_D_6_, Y_6_D_M6_, Y_1_K, K, and Y_1_). Their amphipathic α-helix forming tendencies and their roles in the protective ability of PpDHNA were the subsequent focus. Initial prediction through *in silico* analysis indicated the ability of D-segments to adopt amphipathic α-helices. Experimental confirmation of such prediction was obtained from NMR studies, which showed that the α-helical propensity of D-segment (D_1_) was much higher, as reflected in the Cα and Hα chemical shifts, compared with the shuffled D-segment (D_M1_). Subsequently, CD spectroscopic data further established the ability of the D-segment to exhibit coil-to-helix transition upon addition of TFE. This was absent in the shuffled D-segment, indicating the significant role played by the specific organization of D-segment amino acid residues in inducing the helical backbone. As previously reported, the K-segments in AtHIRD11 and AtLEA4 dehydrins were responsible for promoting helix transition (18, 42). Our study reports a similar α-helix gaining tendency of the D-segments in the presence of TFE.

### PpDHNA activity is size as well as D-segment dependent

The extreme stress-mitigating properties of PpDHNA, especially in the light of the newly identified D-segments, prompted us to investigate high-temperature and desiccation stress mitigation by PpDHNA and its deletion mutants. Especially, we were interested in varying the size and the number of D-segments in the deletion mutants to establish a clear mechanistic role of the D-segment. Experiments were performed *in vitro* (LDH aggregation protection), *in vivo* (proteome protection in *E. coli*), and *in planta* (stress tolerance assay in tobacco).

DHN is known to protect the enzyme LDH from various types of stress (23, 25, 26, 39). The K-segment in DHN plays a major role in LDH protection, as in the case of RcDhn5, where the deletion of two K-segments resulted in a drastic decrease in LDH activity (39). A similar conclusion was drawn from a study with wheat DHN-5 deletion mutant (41). Recent studies with *Arabidopsis* DHN have pointed toward the amphipathic α-helical organization of the K-segment as an important aspect for LDH protection (13, 42). Not just the K-segment, DHN size has also been found to be correlated with the degree of functional protection provided to LDH under stress (23, 39).

Apparently, both mechanisms were found to be operative for the deletion mutants that we studied. For PpDHNA, Y_11_D_11_, and Y_6_D_6_, for which both size and the number of D-segments decreased, there was a concomitant increase in the PD_50_ values. Two variants, Y_6_D_M6_ and Y_6_D_6_, were analyzed that had identical size, amino acid composition, and comparable hydrodynamic radii. The variant containing shuffled D-segments showed a 5-fold higher PD_50_ value than the one containing the wildtype D-segment. This result clearly shows the importance of the D-segment, irrespective of the size of the protein. Of course, size also plays a role as reflected in the PD_50_ values of two variants Y_11_D_11_ and Y_6_D_6_, both containing wildtype D-segments but one double the size of the other with the smaller variant exhibiting a PD_50_ value almost three times that of the larger variant.

It was reported earlier that SbDHN1 could prevent aggregation of proteins under both *in vitro* and *in planta* high-temperature stress conditions (26). A study on wheat dehydrin DHN5 (15) and WZY2 (14) reported the K-segment as the most critical contributor in protecting against various stress-mediated intracellular protein aggregation in *E. coli* cells and *in planta*, respectively. We followed the *in vitro* aggregation protection experiments described above by *in vivo* and *in planta* studies. Stress-induced protein aggregation in *E. coli* cells and tobacco plants was found to be the least for PpDHNA transformed systems. This is how PpDHNA improves the cell viability or decreases the lethal stress damages of the respective organism. The observed degree of protection also correlated with the size and the number of D-segments present in the PpDHNA variants.

Plants transformed with Y_11_D_11_ and Y_6_D_6_ exhibited lower relative electrolyte leakage, thus lowering the relative water loss and consequent lower lipid peroxidation implying lower membrane damage (43). As a first line of defense, in the form of innate immunity, plants commonly produce basal level of osmoprotectants such as sugar and proline. However, under abiotic stress situations, overproduction of these osmoprotectants is considered as an adaptive response defense strategy (44), provided the abiotic stress is not too intense to induce fatal damage. NT and Y_6_D_M6_/Y_1_-transformed tobacco plants failed to survive under extreme stress and were unsuccessful in modifying the osmoregulatory levels. In contrast, under similar levels of stress, the D-segment transformed mutants (PpDHNA, Y_11_D_11_, and Y_6_D_6_) not only were found to be healthy but also exhibited high levels of osmoprotectants indicating that they could launch a successful adaptive response. Of interest, the levels of osmoprotectants produced were found to be correlated with number of D-segments present in the PpDHNA variant in the plant. This showed the direct role played by the D-segments in stress mitigation. The Y or the D_M_ segment-containing variants were incapable of mitigating stress, whereas the K-segment-containing variant did show intermediate stress mitigation. This underscores the common mechanism (α-helix formation under stress) followed by both the K- and D-segments to mitigate stress.

### D-segment-mediated PpDHNA self-association is required for stress abatement

The generally accepted working principle of DHNs is associated with their higher expression under stress conditions and interaction with their partner molecules to function as a molecular chaperone (17). According to the hypothesis by Ingram and Bartels, several K-segments, when present in α-helical conformation, might form intermolecular homobundles (20). In addition to the DHN–DHN interaction, DHNs can also participate in nonspecific interactions with other cellular target proteins to protect the latter from stress-mediated aggregation (17). DHNs are capable of interacting with partly dehydrated surfaces of various other proteins. Such interactions enhance the formation of amphipathic α-helices and prevent the loss of water envelope, leading to irreversible protein denaturation and subsequent aggregations (2). The transiently folded portion of the DHN molecule is generally engaged in intra- and intermolecular binding that is sustained until refolding of the partner protein succeeds (6).

Gel filtration experiments conducted in this study firmly established the existence of self-association mediated by the D-segment in Y_6_D_6_. However, Y_6_D_M6_ failed to self-associate and eluted in later fractions during gel filtration. Although both variants were shown to be associated with comparable hydrodynamic diameter, only Y_6_D_6_ showed self-association, due to the transition toward an α-helix and a simultaneous increase in the size.

The BiFC data, under nonstressed condition, revealed that the DHN/DHN interaction in case of the D- or K-segment bearing PpDHNA (and its deletion mutants) dominates in the membrane region of living plant cells. CD analysis showed the TFE (a membrane mimicker) induced coil-to-helix transition of D-segment bearing variants, in good accordance with BiFC findings. The dimer-associated fluorescence was compatible with the DHN/DHN interaction in the vicinity of cellular membranes where amphipathic α-helices play a significant role. On stress exposure, the dimeric complexes became more fluorescent and were distributed in the cytosol. Therefore, the D-segment variants could not only interact with each other but also respond to applied stress by modulating the degree of interaction and their subcellular distribution. This implies a functional role (molecular shield) played by D-segment-mediated DHN/DHN self-association. This leads to the conclusion that, in addition to the K-segment (8, 9, 16, 45), PpDHNA variants bearing D-segments can also interact with the cellular membrane owing to their amphipathic nature.

Our results are in close agreement with a recent finding where DHN/DHN interaction study in OpsDHN1 revealed the K-segment to be the major driver in inducing the interaction (24). The present study also correlates well with the previous finding from PvLEA6, where it was demonstrated that PvLEA6 could form dimers in living plant cells exhibiting its typical oligomerization property. It was also hypothesized that this could be a probable characteristic of LEA6 for its *in vivo* functionality and possible mode of action (46). Even *Arabidopsis* Cor15a had been reported to form oligomeric complexes that provided cryoprotection to LDH molecules (47).

### Recurring D-segments: implications for stress mitigation in poikilohydric plants

The most important finding of this work is the identification of the D-segment in PpDHNA. We showed that the D-segment can adopt amphipathic α-helical backbone under stress and plays a vital role in stress mitigation, mediated by intermolecular interactions. It should be noted that an earlier study on PpDHNA had pointed out the D-segment, in passing, was another recurring motif as seen in several dehydrins (32). Also, the previous study had identified only 5 (as opposed to 11 as reported here) D-segments in PpDHNA without any experimental or *in silico* studies about its functional importance.

What is the significance of the newly identified D-segment, especially its recurrent occurrence in PpDHNA? Is the D-segment-induced stress mitigation property of PpDHNA unique to *P. patens*? Or did it evolve in certain plant lineages to cope with unique stress? A search of available sequence database of dehydrins and rehydrins showed the appearance of the D-segment in two (almost identical) rehydrin proteins in *S. ruralis* (Tortula). Multiple sequence analysis (Fig. S7) showed the presence of 14 D-segments in the 608 residue rehydrin and 16 D-segments in the 648 residue rehydrin, all appearing just after the appearance of Y-segments. The function of rehydrin is to rejuvenate a plant after almost total desiccation. In that sense, rehydrin is under the most stringent pressure to facilitate extreme stress recovery by plants. The appearance of a large number of D-segments in the rehydrins, larger than the corresponding number (11) in PpDHNA, clearly indicates that the serendipitously discovered D-segments in PpDHNA probably play a key role in extreme stress mitigating in poikilohydric plants. The association between D-segments and plants under extreme abiotic stress was also evident in a recently published whole genome sequence for *Ceratodon purpureus*, a drought-tolerant moss (48). Its genome contains a dehydrin signature-bearing hypothetical protein (KAG0603930.1) containing ten D-segments. The genome contains six isoforms of the protein containing variable number (3–8) of D-segments (Fig. S8).

Now the questions arise, what is the functional advantage of having multiple putative helix-forming D-segments in one chain? Why should it appear contiguous to the Y-segment? Why would it always terminate in the GMGP motif (Figs. S7 and S8)? It is beyond the scope of this work to provide definite answers. We speculate that the D-segment and the Y-segment function in tandem. With two conserved Gly residues, the GMGP motif probably plays a role in terminating the helix. We also propose the following mechanism that can explain why the presence of multiple D-segments may confer a certain functional advantage to dehydrins and rehydrins that have to deal with extreme stress. As shown in this work and elsewhere, a functional requirement of DHN is its self-association, mediated by the D- or the K-segment. In other words, the D-segments must associate with each other under stress. And this has to happen quickly and with strength if the stress is extreme. If the segments occurred only a few times in a single chain, then their association would involve bringing together several chains, which may be slow, and the interactions may be weak owing to the intermolecular nature. On the other hand, if they occurred multiple times in a single chain, then fast and strong intramolecular association would precede any slow and weak intermolecular interactions. Thus, multiple appearance of the D-segment in a single chain would be beneficial to dehydrins and rehydrins that work under extreme stress.

## Experimental procedures

### Plant materials and growth conditions

*P. patens* strain Gransden was received as a kind gift from Prof David Cove, University of Leeds, UK. It was grown on BCDAT media and maintained at a photoperiod of 16 h light and 8 h dark with a photon flux of 100 μmol m^−2^ s^−1^ at 22 °C. Tobacco plants *Nicotiana tabacum* (variety SR1) used in this study were received from Prof Arun Lahiri Majumder, Bose Institute, India, and grown under 16-h photoperiod and 24 °C with 75% relative humidity.

### Bioinformatic analyses

The sequence of full-length PpDHNA protein was analyzed using DISOPRED3 (49) for the presence of probable structurally ordered regions and putative helix-forming amino acid residues, respectively. For the prediction, a cutoff value of 0.5 was used since it yielded accuracy estimates of ∼93.1% with Matthew's correlation coefficient of 0.51 for the false-positive rate threshold of 5% (49). In addition, the Glob Plot 2 server was used to determine the degree of disorder of PpDHNA protein (50). The online program “HeliQuest” (51) was used to analyze the amino acid sequence in peptides capable of forming α-helix (Tables S2 and S4). The LOGO representation of α-helix-forming sequences was generated through the WebLogo program (52).

### Construction, expression, and purification of PpDHNA and its deletion mutants

The coding sequence of PpDHNA (Accession No. AAR13080.1) was amplified from cDNA, prepared by using 1 μg of total RNA as mentioned earlier (33). The full-length coding sequence of PpDHNA was used as a template for generating the deletion mutants using specific primers. The details of the primers used in this study are given in Table S5. The amplified PCR products of deletion mutants (Y_11_D_11_, Y_6_D_6_, Y_6_D_M6_, and Y_1_K) were cloned into a pGEMT-Easy vector (Promega) followed by subcloning into the pET19b expression vector at *NdeI* and *XhoI* sites. The Y_1_ and K deletion mutants were subcloned in pGEX-5X-3 at *EcoRI* and *XhoI* sites. The recombinant proteins were expressed into *E. coli* Rosetta (DE3) pLysS bacterial host strain as described (26). The expressed proteins were analyzed on 12% SDS-PAGE.

The recombinant proteins (Y_11_D_11_, Y_6_D_6_, Y_6_D_M6_, and Y_1_K) were purified using a Ni-NTA column, and the His-tag was removed for the subsequent experiments. The K and Y_1_ deletion mutants with GST tag were purified using glutathione agarose beads, and the tag was removed by on-column digestion with Factor-Xa. The purified proteins were quantified by using the Bradford method (53). Immunodetection analysis was performed as described (33).

### Circular dichroism spectroscopy analysis

CD spectra for PpDHNA and its deletion mutants (Y_11_D_11_, Y_6_D_6_, Y_6_D_M6_, Y_1_K, K, Y_1_, D_1_, and D_M1_) were recorded on a JASCO-1500 CD spectropolarimeter in 50 mM sodium phosphate buffer at 25 °C. Purified PpDHNA and its deletion mutants (Y_11_D_11_, Y_6_D_6_, Y_6_D_M6_, and Y_1_K) proteins without any tag were subsequently dialyzed in 50 mM phosphate buffer pH 7 and used for the CD analysis at a final concentration of 0.4 mg/ml. The HPLC-purified peptides D_1_ (PRQEGIMDKVKNAVGMG) and D_M1_ (PRVQMENKGDIKVAGMG) were purchased from ABclonal Science. The CD scan was performed from 260 to 200 nm at a speed of 100 nm/min with a resolution of 2-nm bandwidth by using a 1-mm quartz cuvette (Hellma). The CD spectra of the proteins in increasing concentration of TFE 10% to 50% were also recorded. These assays were reproduced using proteins from at least three independent purification batches, and the plot shows the average of three independent experiments.

### NMR spectroscopy

NMR experiments for the peptides D_1_ and D_M1_ were carried out on a Bruker Avance III 500 spectrometer. Samples (2 mM) were prepared in 50 mM sodium phosphate buffer (pH: 7.1) at 25 °C containing 10% ^2^H_2_O and the sodium salt of 3(trimethylsilyl) propionic-2,2,3,3-d_4_ acid (internal standard). NMR experiments were performed at 4 °C for spectral clarity owing to the presence of multiple overlapping peaks at higher temperatures. Water signals were suppressed by the standard excitation sculpting procedure. Complete resonance assignments for the two peptides were achieved by using phase-sensitive TOCSY, NOESY, and ^1^H-^13^C (natural abundance) heteronuclear single quantum coherence experiments (Tables S6 and S7). A mixing time of 200 ms was used for the NOESY experiments. NMR data were processed using Bruker Topspin 3.2 and NMRFAM-SPARKY 1.41 software.

### LDH protection assay

Heat inactivation of LDH was assayed as described (26, 39) with some modifications. Briefly, LDH was diluted to a final concentration of 10.5 μg/ml, *i.e.*, 300 nM (monomer) with or without the corresponding additive proteins (PpDHNA, Y_11_D_11_, Y_6_D_6_, Y_6_D_M6_, Y_1_K, K, Y_1_, or BSA) in 10 mM sodium phosphate buffer (pH 7.5). Different additive proteins were mixed in a molar ratio from 1:0.5 to 1:40 (LDH:additive protein), where 300 nM LDH:300 nM additive protein represents a 1:1 molar ratio. A mixture of 100 μl v/v LDH with or without additive proteins (variable molar ratio) was incubated for 10 min at 54 °C. After the stress treatment, the LDH activity was measured by diluting the mixture to 1 ml of reaction mix containing 1.1 mM pyruvic acid and 0.13 mM NADH. Oxidation of NADH was measured by recording the A_340_ for 5 min. Each sample was measured for at least three independent replicates using three independent purification batches of proteins. The data were represented as the percentage recovery of LDH activity *versus* additive protein concentration.

### Aggregation assay

Aggregation assay was carried out to evaluate the role of PpDHNA, its deletion mutants, and BSA (positive control) during high-temperature stress. LDH was subjected to high-temperature stress at 54 °C at different time intervals (10, 20, 30 min) in the presence and absence of PpDHNA, its deletion mutants, and BSA at a 1:4 molar ratio. From a number of different conditions, we selected 54 °C × 10 min as the optimal stress effect. The protein samples were stained with 150 mM Congo red solution. Aggregation of protein was visualized using red filter of the fluorescence microscope (Leica) under 20× objective and measured in a fluorescence spectrophotometer (HITACHI) with excitation at 496 nm and emission at 614 nm. Data from three individual experiments using three independent purified batches of proteins have been analyzed and represented as fold change value of relative fluorescence unit of the samples with respect to unstressed LDH enzyme.

### Cell survivability and proteome protection assay

To determine the high-temperature stress tolerance activity of PpDHNA and its deletion mutants, transformed *E. coli* cultures were induced with 1 mM IPTG at the mid-log phase and after 20 min, the absorbance value was adjusted to equal cell concentration, *i.e.*, Ab_600_ ∼ 0.6 (1.6 × 10^8^ CFU/ml). In order to evaluate the expression of individual recombinant proteins, immunoblot detection was performed using an anti-HIS antibody, taking an equal amount of protein (250 μg). Then the cells were incubated at 54 °C for another 20 min to impart high-temperature stress. The samples were spread on the LB agar plates containing ampicillin (100 mg/l) and chloramphenicol (34 mg/l) antibiotics. NT *E. coli* Rosetta (DE3) pLysS bacterial host strain was kept at 37 °C (unstressed) and another set at 54 °C heat stress for 20 min. The colony-forming unit (CFU) was calculated after 16 h of incubation at 37 °C, and the survivability rate was represented as a percentage of cell survivability compared with the unstressed cells.

Furthermore, stress-treated bacterial cells overexpressing PpDHNA and mutant proteins were harvested. The cell pellets were resuspended in sonication buffer containing 20 mM Tris-HCl, pH 7.5, 1 mM EDTA, 1% TritonX-100, 10 mM β-mercaptoethanol, and 2 mM PMSF followed by sonication (Hielscher) on ice with 80% amplitude for 15 s. Total protein was quantified using the Bradford method (53); 250 μg total protein sample was also considered for subsequent analysis. Upon centrifugation (3000*g* for 10 min), the amount of protein present in the supernatant and pellet fractions was quantified using the Bradford method (53). An equal volume of the supernatant and the pellet fractions (after resuspending the pellet in the same volume of sonication buffer as that of the supernatant fraction) was analyzed on a 12% SDS PAGE to visualize the relative differences in their proteomic profile upon heat stress exposure. Stress-treated *E. coli* Rosetta (DE3) pLysS bacterial host strain was used as a control. The experiment was repeated thrice with three experimental replicates.

### Confocal microscopy and fluorescence emission spectroscopy of *E. coli* cell aggregates

PpDHNA and its deletion mutants transformed *E. coli* cells were induced for 2 h with 1 mM IPTG at mid-log phase and the absorbance values were adjusted to identical cell concentrations. Cultures were subjected to high-temperature stress at 54 °C for 20 min and washed with PBS buffer, followed by staining with Proteostat dye (Enzo Life Sciences). The cells were then observed under the TRITC filter of confocal microscope (Olympus IX81; 40× objective). Absorbance was also measured in the spectrofluorometer at an excitation of 550 nm and an emission of 603 nm. NT *E. coli* Rosetta (DE3) pLysS bacterial host strain exempted from stress were grown at 37 °C (unstressed) and another high-temperature stress-treated set served as a control. The experiment was repeated twice, and the data were represented as fold change value of the relative fluorescence unit of the samples with respect to unstressed cells.

### Generation of overexpression lines of PpDHNA and its deletion mutants

The *PpDHNA* gene and its deletion mutants were subcloned in the pCAMBIA1301 binary vector at *Xba*I and *Bam*HI sites. The pCAMBIA1301 vector, a gift from Prof. Arun Lahiri Majumder, was modified in our laboratory by cloning GFP at *Sma*I (single site clone) and CaMV35S at *Hind*III and *Xba*I sites. The schematic representation of the modified pCAMBIA1301 expression cassette is shown in Fig. S9. The expression of the introgressed sequence is controlled under the CaMV35S promoter and NOS terminator, where GFP translates as a fusion protein. The pCAMBIA1301-GFP was used to generate vector-transformed plants in this study. The positive constructs for *PpDHNA* and all the deletion mutants were mobilized into *Agrobacterium tumefaciens* strain LBA4404 using the freeze-thaw method (54).

*Agrobacterium-*mediated tobacco transformation was carried out as described (55). The transformation was carried out with leaves from 1-month-old tobacco plants, and MS medium (Murashige and Skoog) supplemented with BAP (2 mg/l), NAA (0.2 mg/l), cefotaxime (250 mg/l), and hygromycin (20 mg/l) was used as the regeneration medium. The plants regenerated from *in vitro* transformation were considered as T_0_ plants (heterozygous for the insertion). For the generation of T_1_ transformed lines, the T_0_ plants were selfed and all the seeds were germinated in MS medium supplemented with hygromycin (20 mg/l).

### Analysis of putatively transformed plants

Genomic DNA was isolated as described (56), and all the putatively transformed plants were PCR screened for transgene and selection marker (*hpt*) (Figs. S10 and S11). Details of the oligonucleotide primers used are given in the Table S5. In order to determine the expression of *PpDHNA* and its deletion mutant-transformed plants, quantitative real-time PCR and Western blot analysis were performed. The pCAMBIA1301 binary expression cassette contains the GFP protein fused with the DHN sequences in each case and is translated as GFP-fusion protein (shown in Fig. S9). Thus, the transcript level of GFP was checked for all the transformed plants. *Actin* was used as an internal control and the relative expression level was calculated using the 2^−ΔΔ*C*T^ method (57). The primer sequences used for the qRT-PCR experiment were designed by Primer3 software and listed in the Table S5. For further confirmation, immunoblot analysis of transformed plants was performed using an anti-GFP antibody. Experiments were performed for at least three independent lines.

### Abiotic stress treatment

For abiotic stress experiments, the T_1_ transformed tobacco plants overexpressing *PpDHNA* and its deletion mutants along with VT lines seeds were germinated in MS medium supplemented with hygromycin (20 mg/l). NT tobacco seeds were germinated in basal MS medium, and all the tobacco plants (NT, VT, transformed plants overexpressing *PpDHNA* and its deletion mutants) were grown for 30 days following the condition as mentioned in the previous section. These plants were transferred to Soilrite and grown for 21 days, initial 14 days with watering and then 7 days without water. At the 7 days water withheld condition, the Soilrite relative water content was measured around 80% (considering 100% soil water content in the wetted condition during watering) before stress treatment. Three randomly selected lines were subjected to high-temperature (32–46 °C) along with desiccation stress for 14 days at 12 h light and 12 h dark with a photon flux of 100 μmol m^−2^ s^−1^ at 65% relative humidity. High-temperature stress was subjected according to the stress regime as shown in Fig. S12. The stress regime was repeated each day and continued for 14 days on a stretch without any watering to generate high-temperature coupled with desiccation stress. On high-temperature coupled with desiccation stress, the relative Soilrite water content was found to gradually decrease to 30% in 14 days. For recovery, the stress-treated plants were placed in normal growth conditions followed by watering. The plants were assayed before and after subjecting to stress treatment for their growth parameters. Plants not subjected to any stress served as a control. All the assays were performed with three randomly selected plant lines of each transformed protein, and single lines from each transformed protein were shown as their representative images.

### Estimation of growth parameters

The plants were assayed before and after subjecting to high-temperature and desiccation stress. The amount of total chlorophyll content in leaves was measured as described (58).

The total soluble sugar content was determined by the anthrone method (59) using glucose as a standard and expressed as mg/g dry weight of leaves.

The proline content was estimated as described (60) and determined from the standard curve and calculated on a fresh weight basis.

Lipid peroxidation in leaves was measured by the determination of MDA content as described (61).

Relative electrolyte leakage was measured to evaluate membrane damage. The upper third, fourth, and fifth positional fully expanded leaves were excised from the plants, and the relative electrolyte leakage was measured as described (33). Similarly, for measuring relative water loss, the leaves were dehydrated at 25 °C in a blotting paper. The weight of the excised leaves was taken at different time intervals of 0, 2, and 4 h. Relative water loss was calculated as a percentage of final weight to initial weight.

### *In planta* protein aggregation assay

The transformed plants along with NT and VT plants were exposed to high-temperature (48 °C) for 4 h. The leaf peels were prepared from the third fully expanded leaf from each transformed plant after stress treatment and stained with 150 mM Congo red solution. The peels were observed under the TRITC filter of a confocal microscope (Olympus IX81; 40× objective). Congo red fluorescence was quantified using ImageJ/Fiji software. The fluorescence was measured in terms of the mean gray value of at least three microscopic fields from each plant line and consecutively normalized to the unstressed tobacco plants. Data were represented as a fold change in relative fluorescence level with respect to unstressed plants. In addition, to avoid the potential effect of metabolites, the aggresomes were isolated from leaf tissues using aggresome isolation buffer (50 mM Hepes pH 7.5, 150 mM NaCl, 1% Triton X-100). Samples were homogenized and centrifuged at 1000*g* for 10 min. The supernatant containing the aggresomes was collected, stained with 150 mM Congo red solution, and observed under a 40× objective of the fluorescence microscope (Leica, red filter). Data are representative of three independent experiments.

### Bimolecular fluorescence complementation assay

For the BiFC assay, YFP^N-ter(1-173aa)^ from pUC-SPYNE and YFP^C-ter(156-239aa)^ from pUC-SPYCE were cloned into pCAMBIA1301 vector individually at the *Bam*HI*/Sac*I site. Thereafter, full-length *PpDHNA* and its deletion mutants were separately subcloned into pCAMBIA1301-YFP^N-ter^ and pCAMBIA1301-YFP^C-ter^ vector at *Xba*I*/Bam*HI site. The expression cassettes of the pCAMBIA1301 constructs have been shown in Fig. S13. The positive clones were mobilized into *A. tumefaciens* strain LBA4404. *In planta* transient transformation in onion bulb was performed as described (62). After 48 h of transformation, the epidermis of the infected zone was peeled and mounted with water before visualization under a YFP filter of a confocal microscope (Olympus IX81; 20× objective). Calcineurin B–like protein 1 (CBL1) and its receptor CIPK24, which interact at the plasma membrane (63), served as a positive control. Coexpressed pCAMBIA1301-YFP^N-ter^ and pCAMBIA1301-YFP^C-ter^ were used as a negative control. From three independent experimental images, YFP fluorescence was quantified using ImageJ/Fiji software. The fluorescence was measured in terms of the mean gray value of at least three individual fields derived from three individual experiments and consecutively normalized to the negative control. Data were represented as a fold change in relative fluorescence level with respect to the negative control.

### Determination of self-association characteristic of Y_6_D_6_ and Y_6_D_M6_

For the purpose of analyzing the size distribution of Y_6_D_6_ and Y_6_D_M6_ proteins, dynamic light scattering experiments were carried out using Zetasizer Nano ZS90 Malvern Instruments (4 mW, He-Ne laser, λ= 632.8 nm) at 25 °C in 50 mM sodium phosphate buffer pH 7. Equal concentration (15 mM) of protein samples were passed through a 0.22-μm filter, before scanning. The hydrodynamic diameter of the samples was calculated using the equation *d*_H_ = (*k*_B_*T*)/(3*πηD*), in which *k*_B_, *η*, and *D* are the Boltzmann constant, viscosity, and translational diffusion coefficient, respectively, at temperature *T*.

Size exclusion chromatography was carried out to determine the self-association property of Y_6_D_6_ and Y_6_D_M6_. The gel filtration column was packed with superose 12 prep grade beads (Sigma-Aldrich). Bacterially overexpressed, purified proteins of Y_6_D_6_ and Y_6_D_M6_ were loaded (2 and 4 mg) onto the column. The proteins were eluted with 50 mM sodium phosphate buffer pH 7 in the presence and absence of 20% glycerol at a flow rate of 0.8 ml min^−1^. The absorbance value of every fraction was taken at 215 nm and plotted against the eluted fraction volume.

CD experiment was carried out to analyze the α-helix formation ability of both the proteins (Y_6_D_6_ and Y_6_D_M6_) in the presence of 20% glycerol (helix-inducing agent) (18). Details of the experimental setup and conditions have been followed as mentioned earlier. All the experiments were performed in triplicate using three independent purified batches of protein.

The effect of high-temperature stress treatment on the self-interaction of both the proteins (Y_6_D_6_ and Y_6_D_M6_) was analyzed through the BiFC experiment. The *Agrobacterium* strain (LBA 4404) harboring Y_6_D_6_-YFP^N-ter^/Y_6_D_6_-YFP^C-ter^ and Y_6_D_M6_-YFP^N-ter^/Y_6_D_M6_-YFP^C-ter^ constructs (as shown in Fig. S13) were transiently transformed in 28-day-old *N. tabacum* leaves as described in the previous section. After 48 h of transformation, transiently transformed plants were subjected to high-temperature stress (48 °C) for 4 h. The leaf peels of stressed and nonstressed plants were observed under the 40× objective of the fluorescence microscope (Olympus IX71). YFP^N-ter^/YFP^C-ter^ constructs were used as a negative control. YFP fluorescence was quantified using ImageJ/Fiji software in a similar way as mentioned previously.

### Statistical significance

All the experiments have been repeated three times individually. Individually each experiment was carried out in triplicates, and data have been represented as means ± SD. Statistical significance was determined using GraphPad Prism 6.0 software, using one-way analysis of variance (ANOVA) followed by Dunnett's multiple comparison test in all the experiments except Figure 8*F*, wherein Tukey-HSD multiple comparison tests were used. In the case of Dunnett's multiple comparison test, significant differences with the control (or unstressed or NT or negative control) set of data have been represented as ∗*p* < 0.01, ∗∗*p* < 0.001, and ∗∗∗*p* < 0.0001, whereas in the case of Tukey-HSD, the different alphabetical letters represent significant differences (*p* < 0.01), and the same letters represent no significant differences.

## Data availability

All data are contained in the article and or supporting information.

## Supporting information

This article contains supporting information.

## Conflict of interest

The authors declare that they have no conflicts of interest with the contents of this article.
