# Supplementary material for: Multiple copies of a novel amphipathic α-helix forming segment in Physcomitrella patens dehydrin play a key role in abiotic stress mitigation
Source: J Biol Chem. 2021 Mar 26;296:100596. doi: 10.1016/j.jbc.2021.100596 (PMC8100072; doi:10.1016/j.jbc.2021.100596)
Supplement: Supplementary file 1 — Figures S1 to S13 [file mmc1.docx]

**Multiple copies of a novel amphipathic α-helix forming segment in *Physcomitrella patens* dehydrin play a key role in abiotic stress mitigation**

Gouranga Upadhyaya^1^, Arup Das^1^, Chandradeep Basu^2¶^, Tanushree Agarwal^1¶^, Chandra Basak^1^, Chandrima Chakraborty^1^, Tanmoy Halder^1^, Gautam Basu^2^, Sudipta Ray^1^*


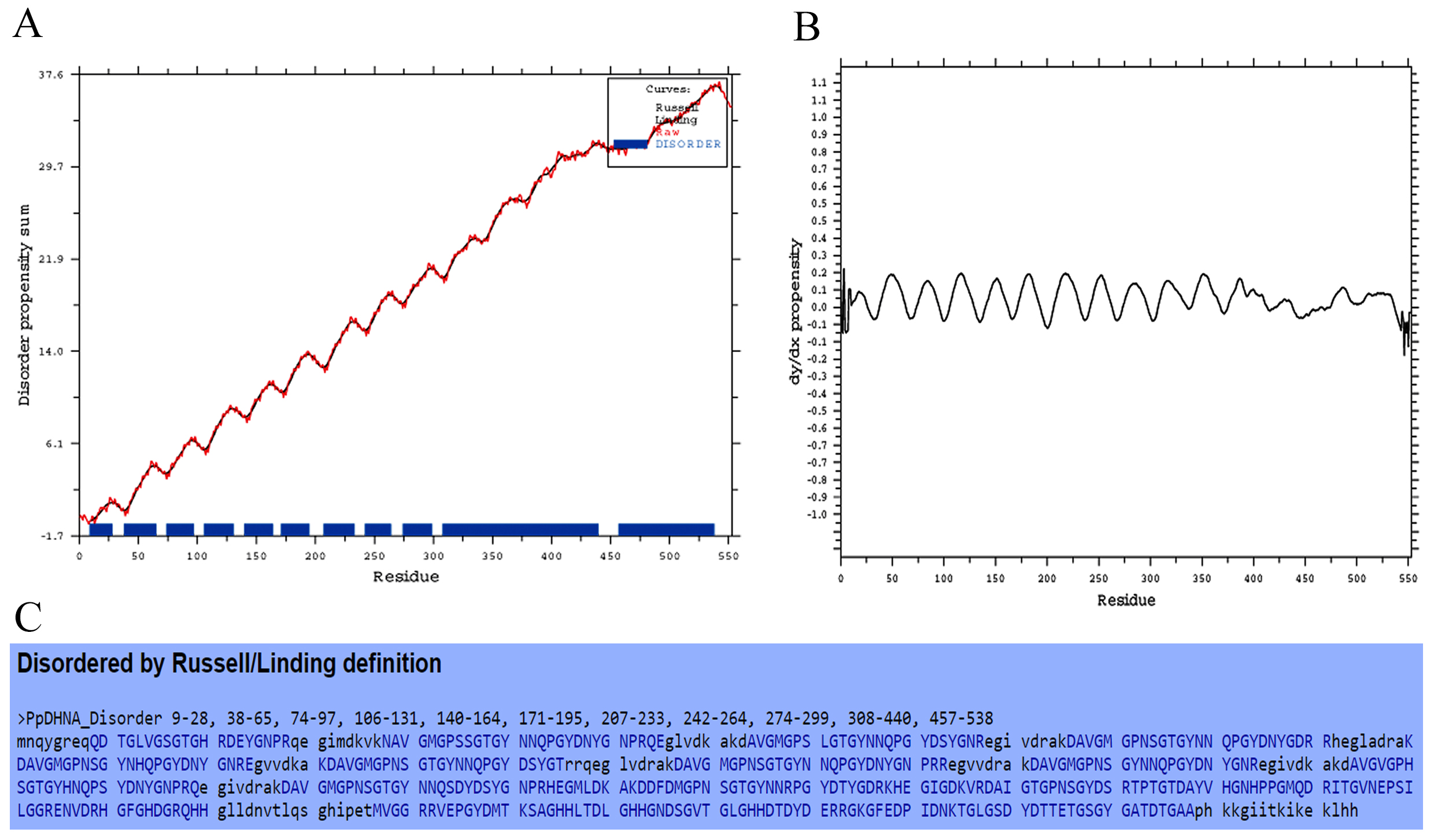


**S1 Figure.** Sequence prediction of PpDHNA for disordered regions by using Glob plot 2 server. (A) The disorder propensity score was plotted against residue number where residue ranges predicted for disordered segments were indicated by thick blue bars. (B) The prediction score was plotted against residue number considering more higher value as more disordered and lower value as less disorder in respect to threshold dy/dx scale 0. (C) Output of analysed PpDHNA sequence showing predicted disordered region by Russell/Linding scale where blue coloured residues were predicted as disorder region. The propensity of a given amino acid to be in “random coil” or in regular secondary structure is determined using the “Russell/Linding” scale, where Propensity of Disorderness (*P)=RC-SS*; RC and SS are the propensity for a given amino acid to be in ‘random coil’ and regular ‘secondary structure’ respectively.


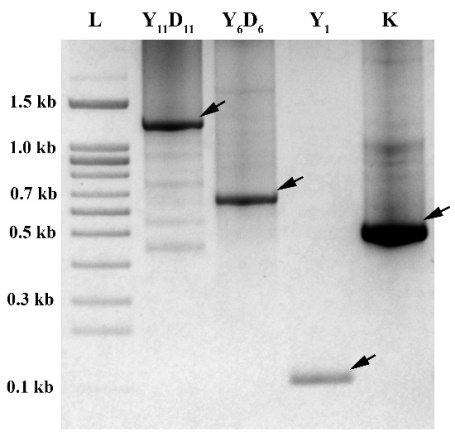

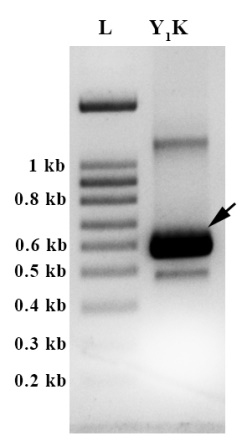


**S2 Figure.** PCR amplification of deletion mutants (Y_11_D_11_, Y_6_D_6_, Y_1_K, K and Y_1_) using *PpDHNA* as template DNA. L represents low molecular weight DNA ladder. Arrow head represent the corresponding amplified bands.


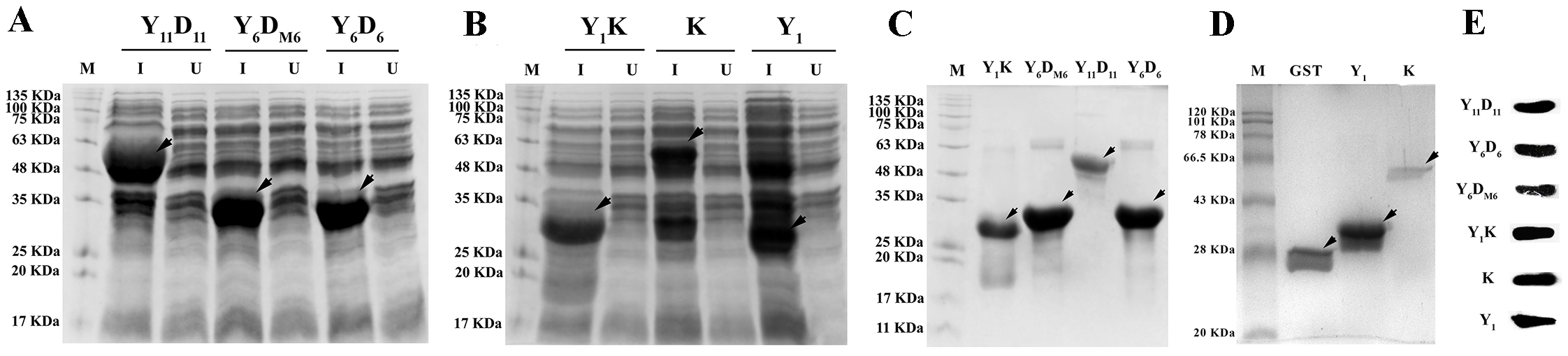


**S3 Figure.** Expression, purification and immunoblot of PpDHNA and its deletion mutants (Y_11_D_11_, Y_6_D_6_, Y_6_D_M6_, Y_1_K, K and Y_1_). (A) SDS-PAGE showing the expression pattern of deletion mutants Y_11_D_11_, Y_6_D_6_, Y_6_D_M6_. (B) SDS-PAGE showing the expression pattern of deletion mutants Y_1_K, K and Y_1_. The molecular weight marker represented with (*M*), and uninduced cells (*U*) and induced cells (*I*). (C) SDS-PAGE showing the NiNTA column purified proteins for deletion mutants Y_11_D_11_, Y_6_D_6_, Y_6_D_M6_, Y_1_K. (D) SDS-PAGE showing the GST agarose column purified proteins for deletion mutants K and Y_1_. The corresponding bands of proteins (K and Y_1_) appeared as GST fusion protein. The molecular weight marker represented with *(M)* and the corresponding bands are marked with arrow head. Purified GST was shown in the SDS-PAGE as a control. (E) Immunoblot analysis for purified proteins with anti-HIS antibody in case of Y_11_D_11_, Y_6_D_6_, Y_6_D_M6_, Y_1_K and with anti-GST antibody in case of K and Y_1_ mutant proteins.


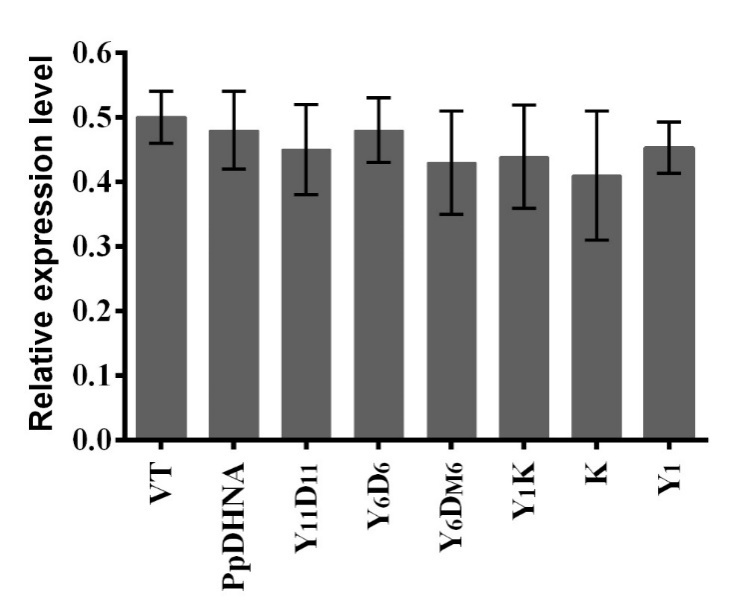

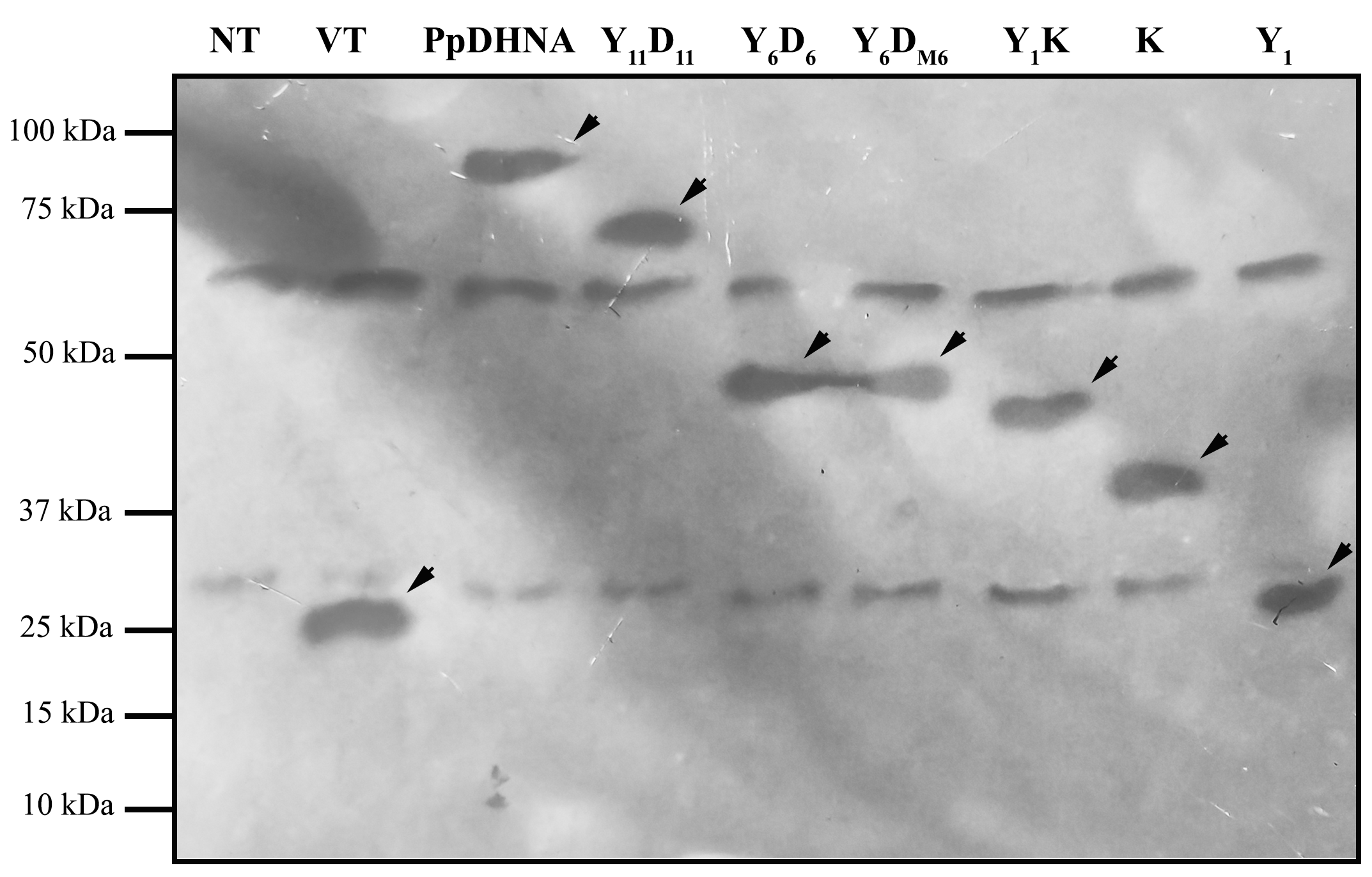
 **A B**

**S4 Figure.** Molecular analysis of *Nicotiana tabacum* plants transformed with PpDHNA and its deletion mutants (Y_11_D_11_, Y_6_D_6_, Y_6_D_M6_, Y_1_K, K and Y_1_) along with non-transformed (NT), vector transformed (VT) plants. (A) qRT-PCR analysis in putative transformed plants for the presence of GFP transcript. *Actin* was used as an internal control and data represented as relative expression level with respect to the *Actin*. Data shown are illustrative of at least three independent replicates. (B) Immunoblot analysis of transformed *N. tabacum* plants using anti-GFP antibody. Specific immunoreactive bands are marked with black arrow head in their respective lanes.


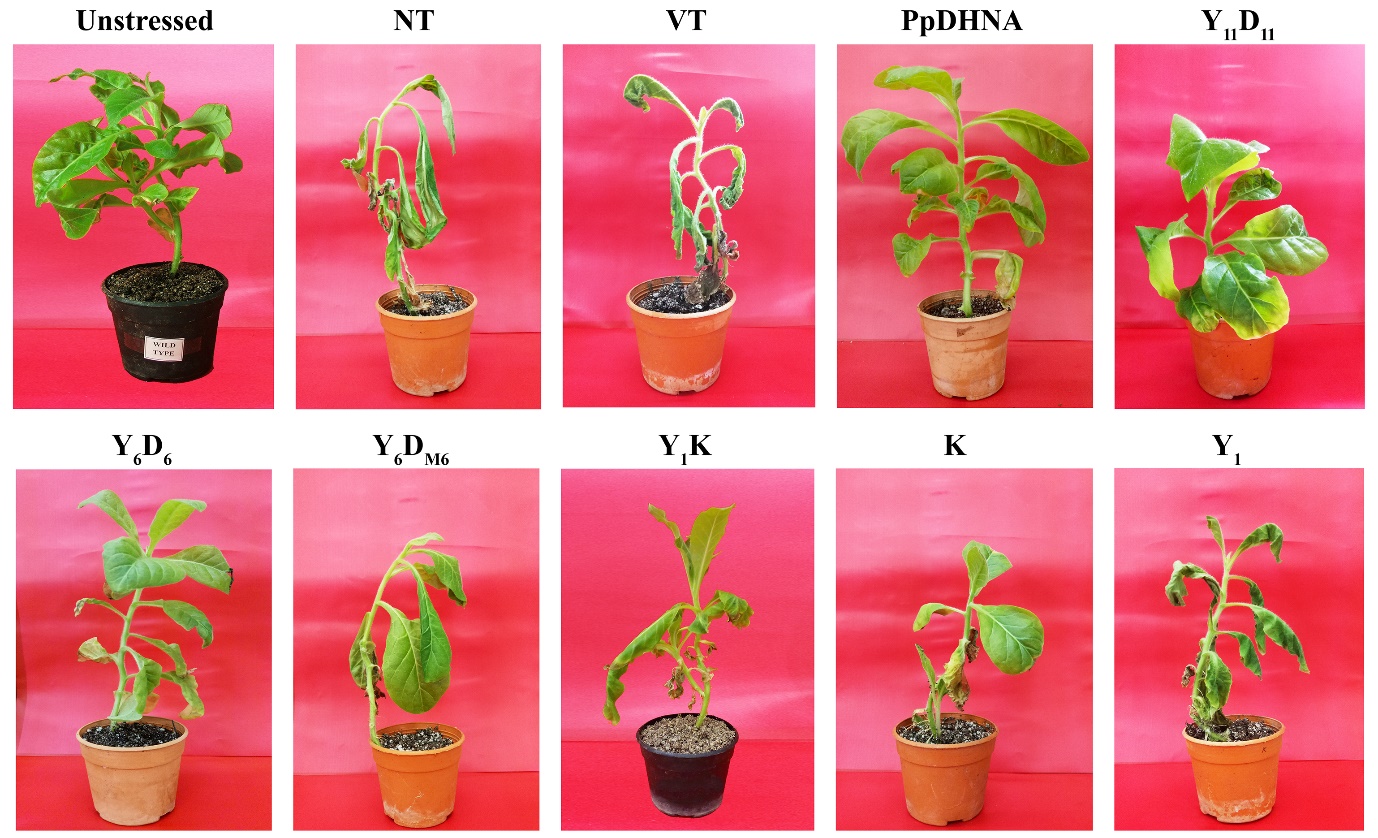


**S5 Figure.** Representation of PpDHNA and its deletion mutants (Y_11_D_11_, Y_6_D_6_, Y_6_D_M6_, Y_1_K, K and Y_1_) transformed *Nicotiana tabacum* plants along with non-transformed (NT), vector transformed (VT) plants after 15 days stress treatment of high temperature and desiccation stress.


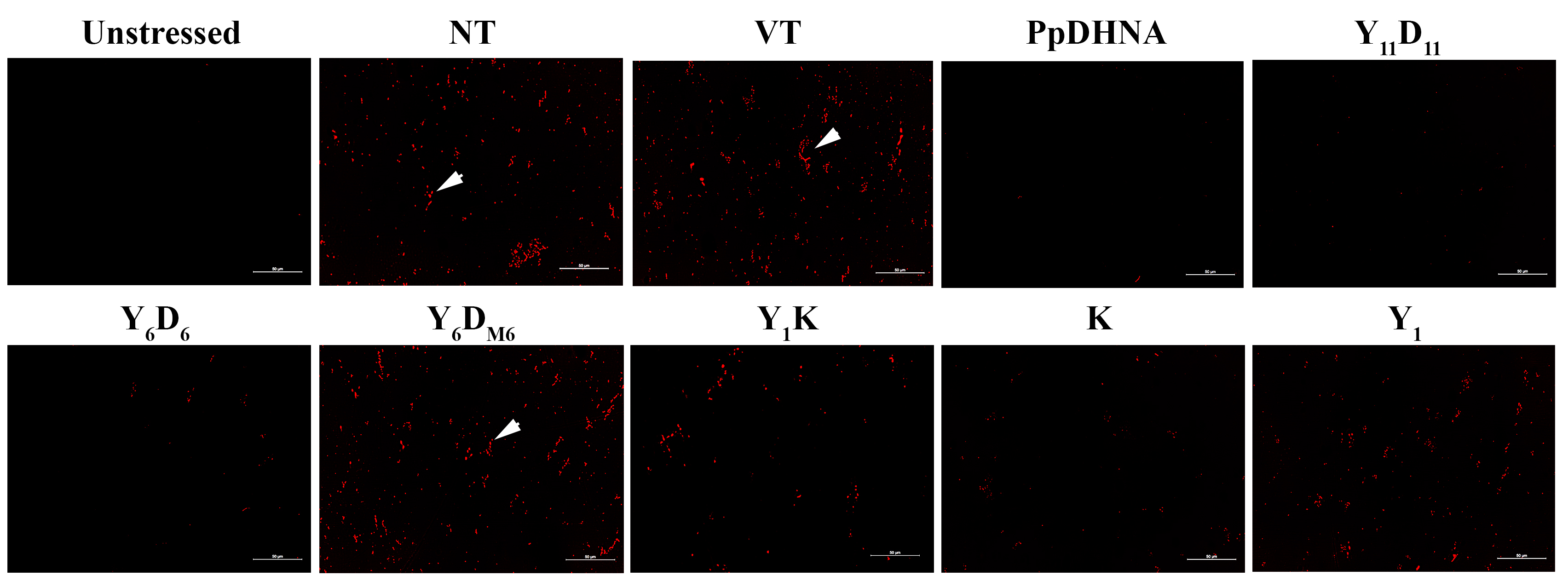


**S6 Figure.** Fluorescence microscope images of isolated protein aggresomes from 4 h stress treated leaves of PpDHNA and deletion mutants transformed *Nicotiana tabacum* plants along with NT and VT plants. Isolated aggresomes were stained with Congo red and observed under red filter at 40X objective; marked with white arrowheads. Scale bars, 20 µm.


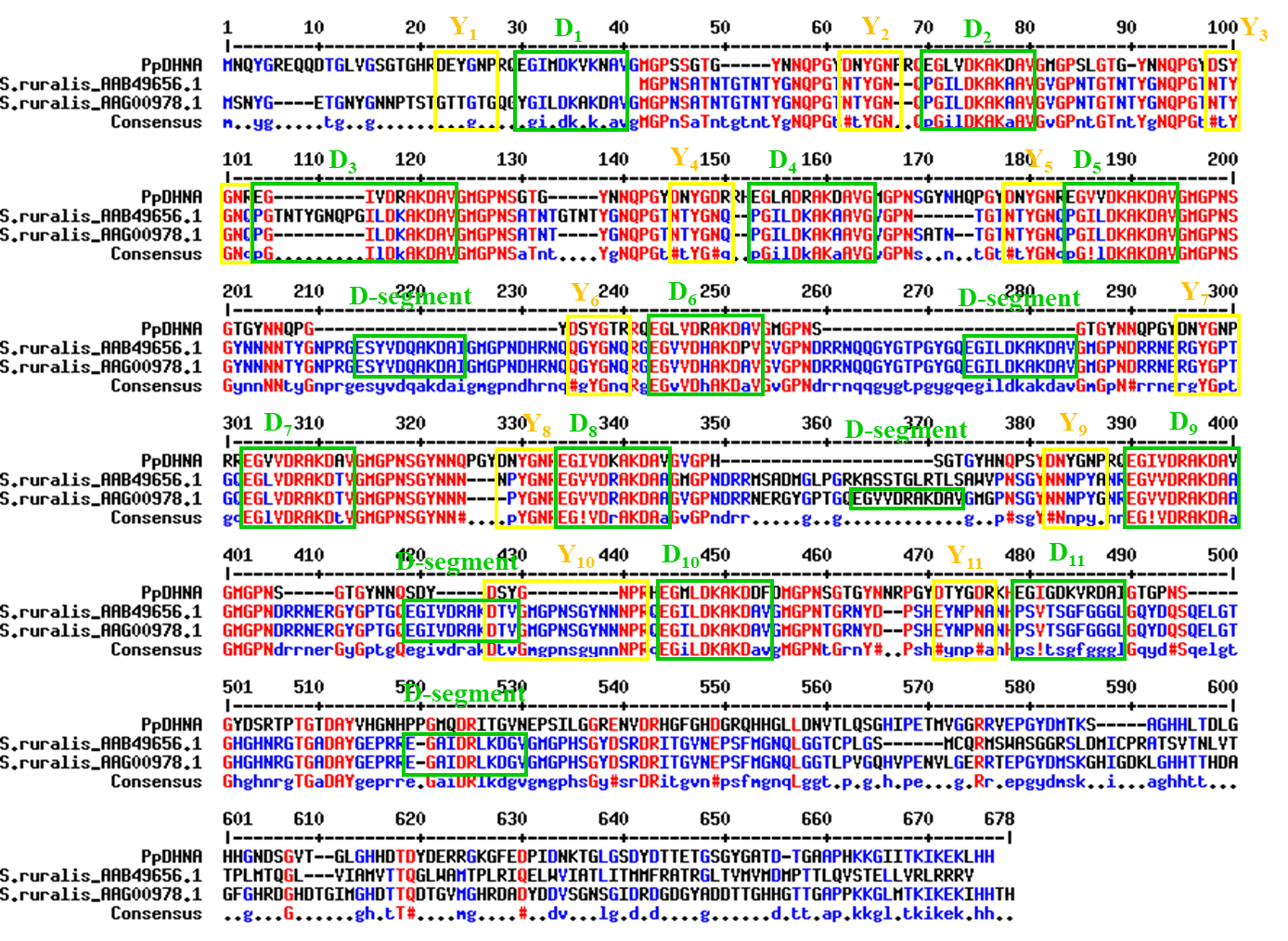


**S7 Figure.** Multiple sequence alignment showing the differences and similarities between PpDHNA protein and *Syntrichia ruralis* rehydrin proteins. Sequence alignment was performed using MultAlin server (<http://multalin.toulouse.inra.fr/multalin/>) where high consensus coloured in red, low consensus coloured in blue and neutral coloured in black. Y-segments are filled with yellow boxes and numbered as Y_1_-Y_11_, D-segments are marked with green boxes denoted with D_1_-D_11_. Additional D-segments found in *Syntrichia ruralis* rehydrin protein sequences are marked as ‘D-segment’ only.


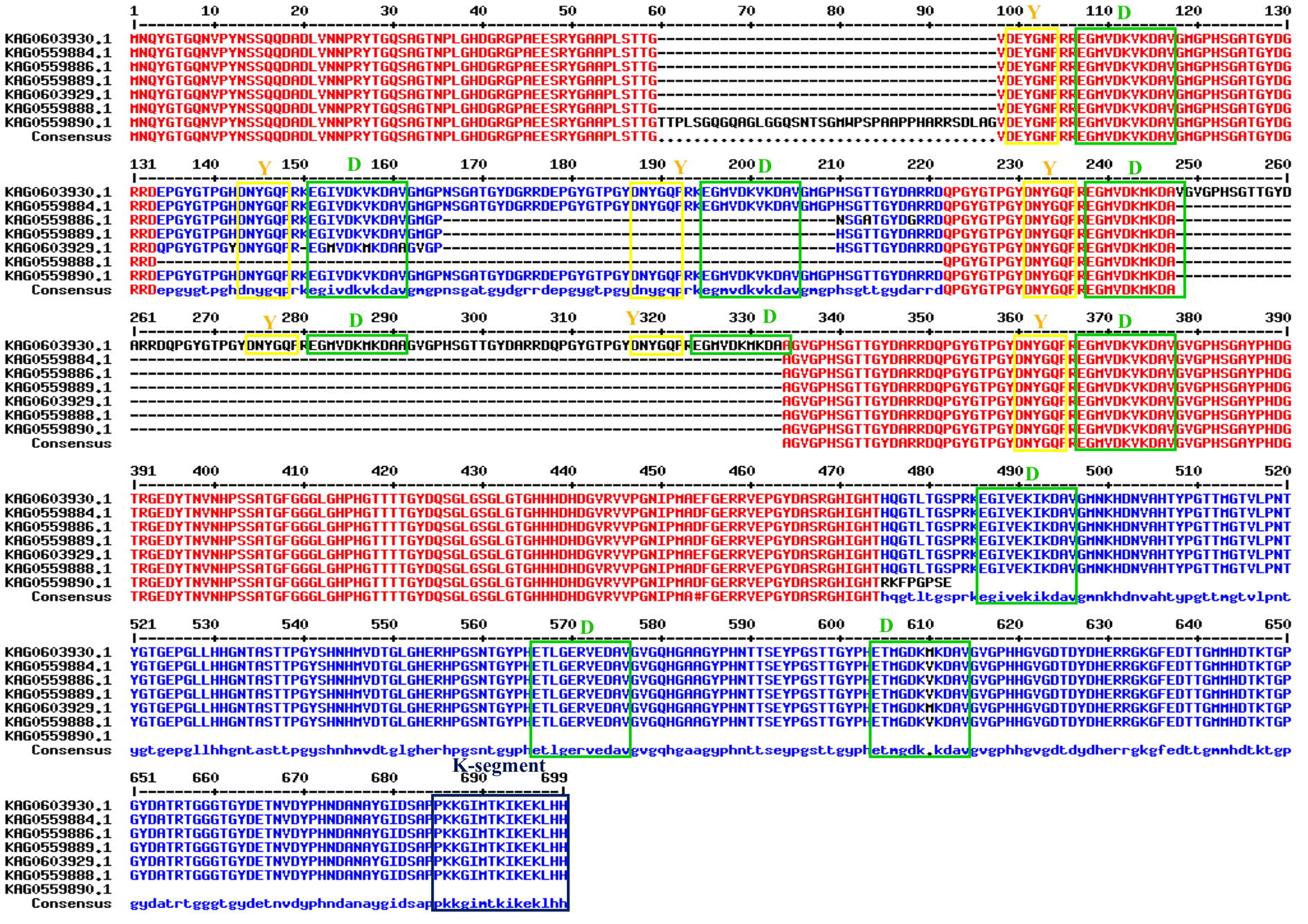


**S8 Figure.** Multiple sequence alignment of a putative dehydrin (KAG0603930.1) and its isoforms from *Ceratodon purpureus*. Sequence alignment was performed using MultAlin server (<http://multalin.toulouse.inra.fr/multalin/>) where high consensus coloured in red, low consensus coloured in blue and neutral coloured in black. Y-segments are highlighted with yellow boxes, D-segments are marked with green boxes and the conserved C-terminal K-segments are marked by black box.

**
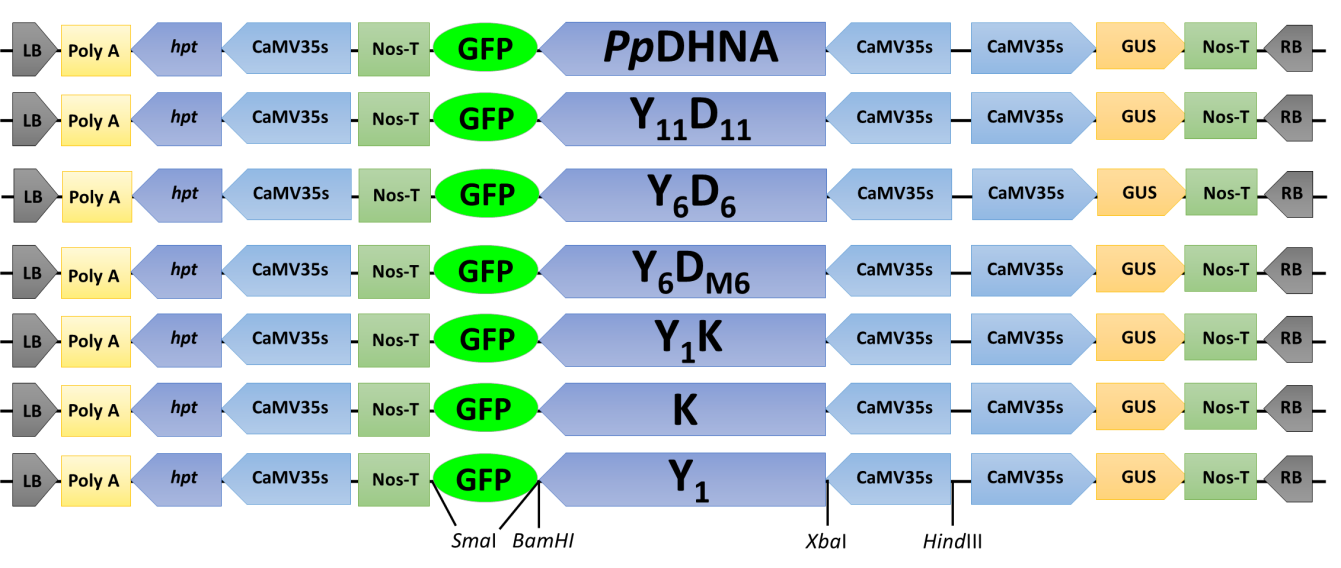
**
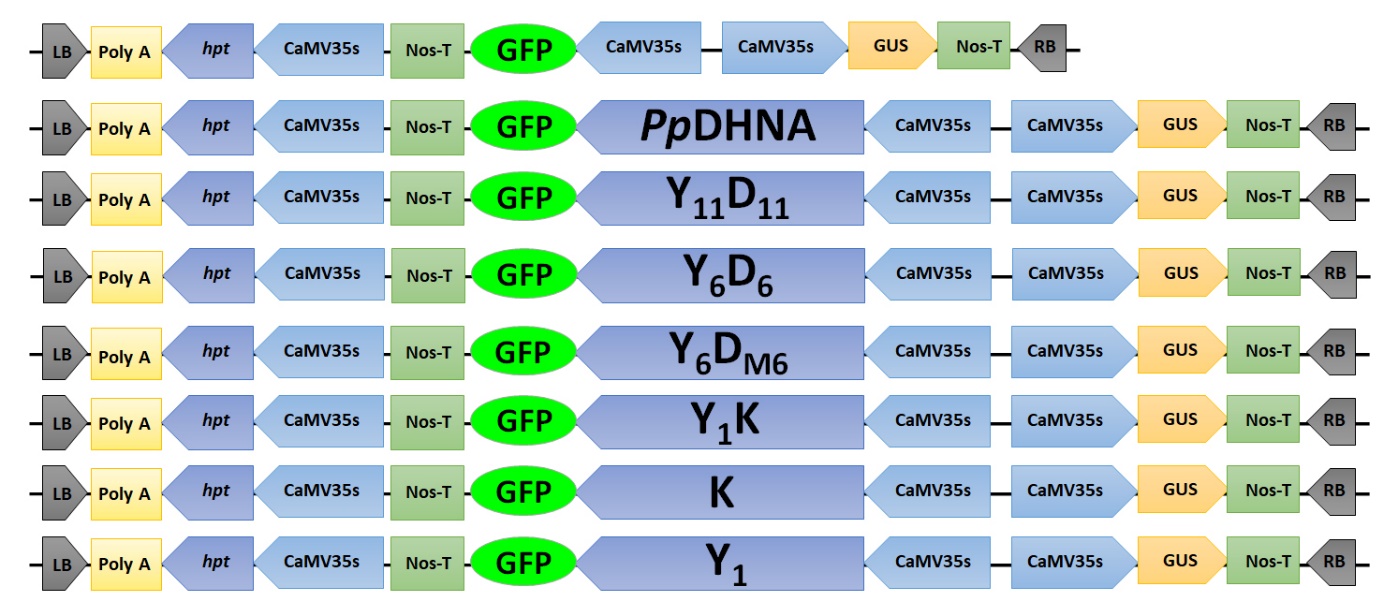


**S9 Fig****ure.** Schematic representation of expression cassettes in pCAMBIA1301 vector used for *Agrobacterium*-mediated *Nicotiana tabacum* transformation.


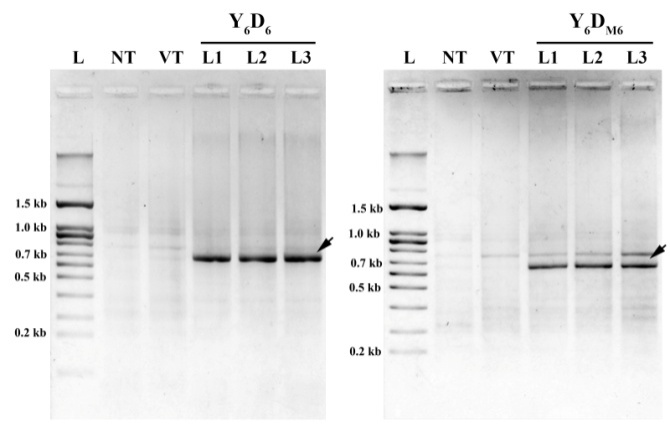
**
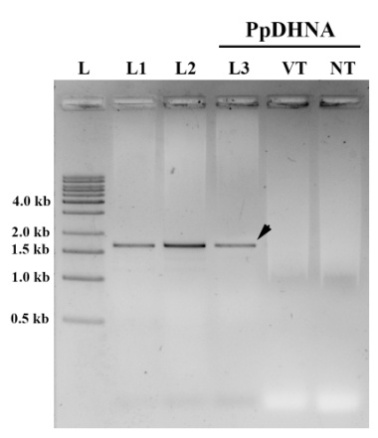

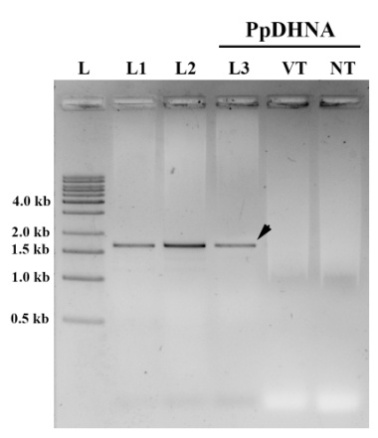
**

**
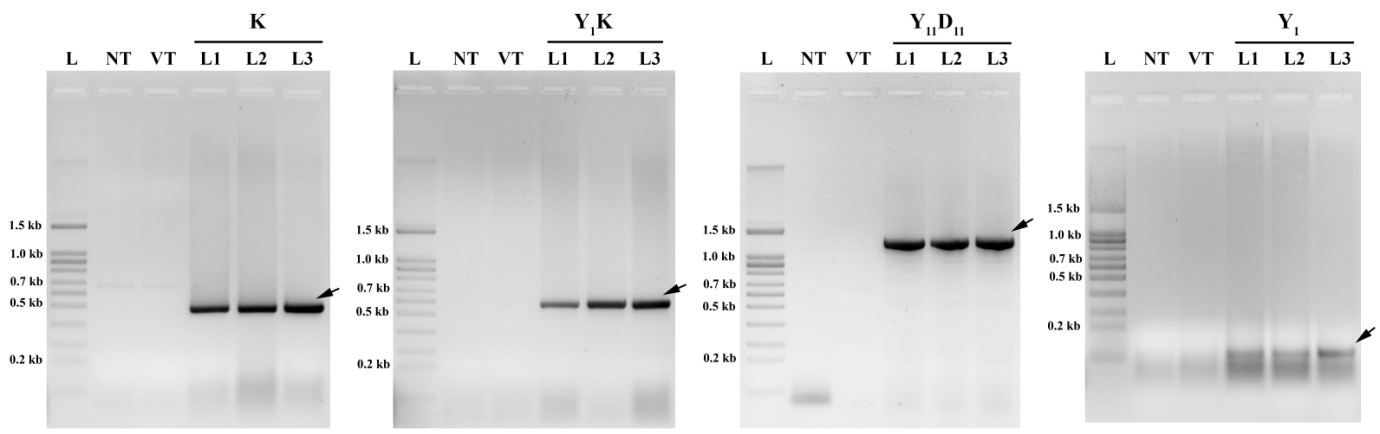
**

**S10 Figure.** PCR amplification of the introgressed sequences using isolated DNA from the transformed lines of *PpDHNA* and its deletion mutants (Y_6_D_6_, Y_6_D_M6_, K, Y_1_K, Y_11_D_11_ and Y_1_) along with NT and VT plants for inserted genes. L represents the low molecular weight DNA ladder. L1-3 represents different independent transformed lines. Arrow head represent the corresponding amplified bands.

**
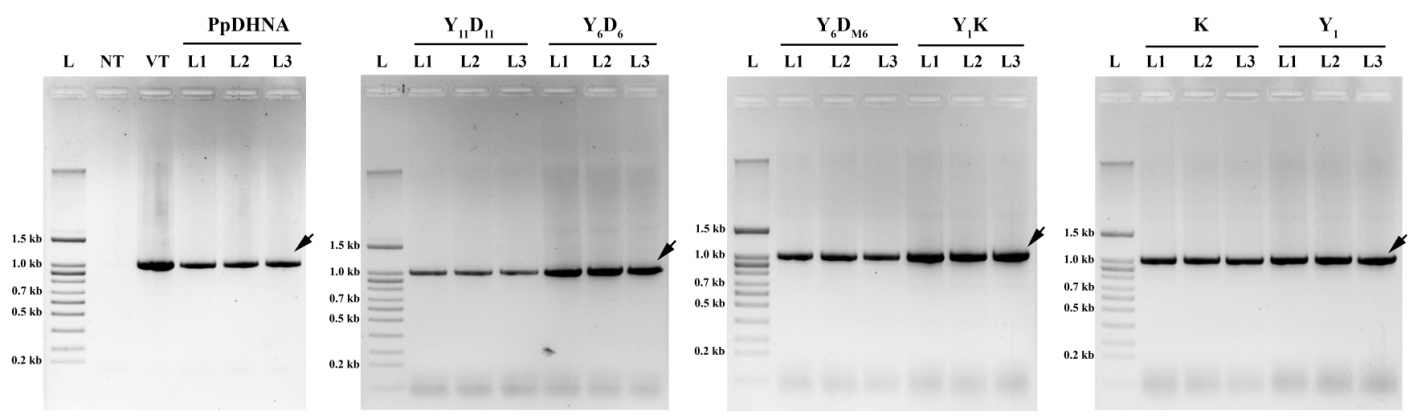
**

**S11 Figure.** PCR amplification of the *hpt* gene using isolated DNA from the transformed *Nicotiana tabacum* lines of *PpDHNA* and its deletion mutants (Y_11_D_11_, Y_6_D_6_, Y_6_D_M6_, Y_1_K, K and Y_1_) along with NT and VT plants for inserted genes. L represents the low molecular weight DNA ladder. L1-3 represents different independent transformed lines. Arrowhead represents the corresponding amplified bands.


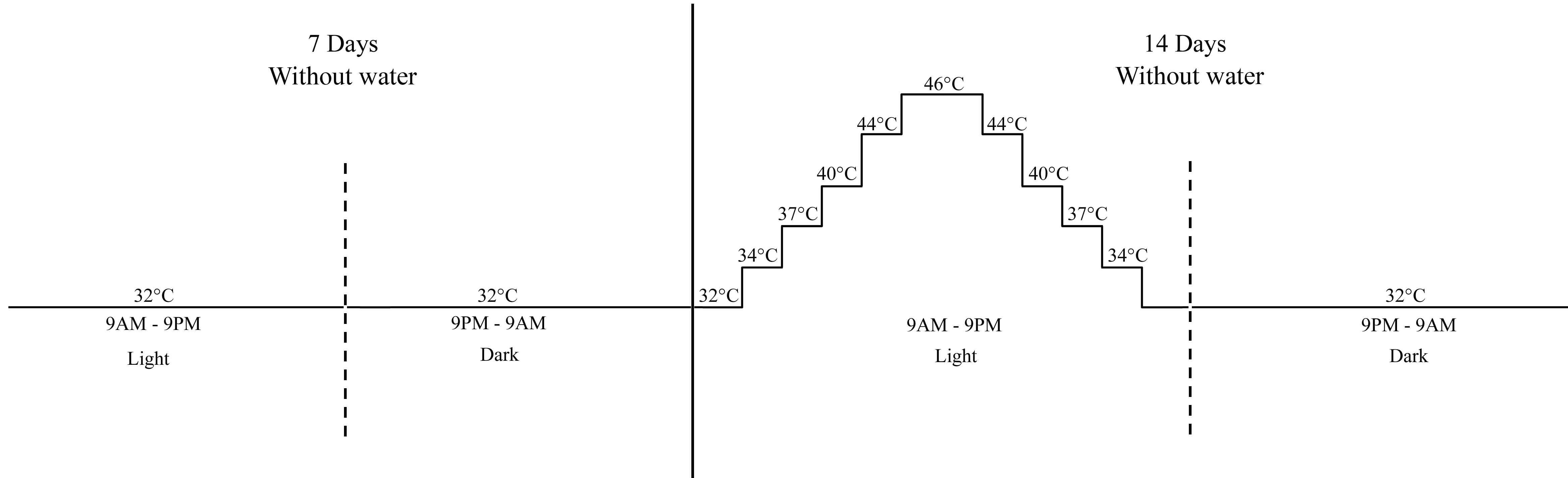


**S12 Figure.** Stress regime under which the transformed lines, VT and NT plants were kept for 15 days with photon flux of 100 μmol m^-2^ s^-1^ and 60% relative humidity.


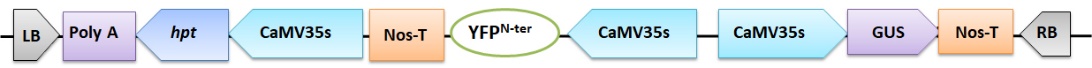
**
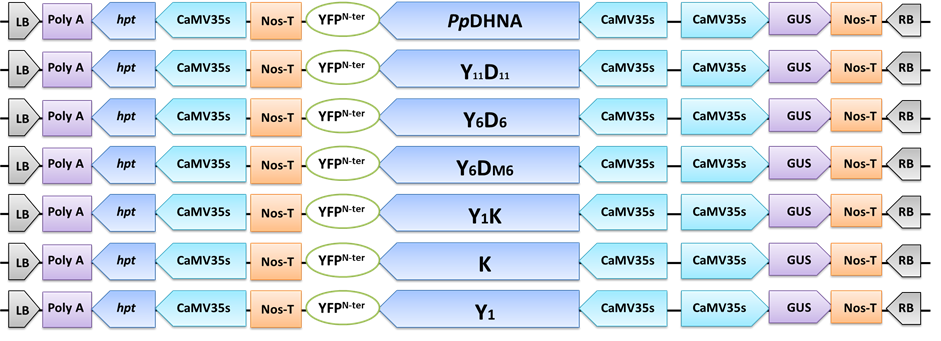
**


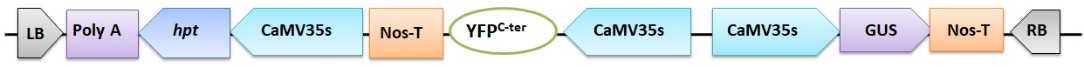
**
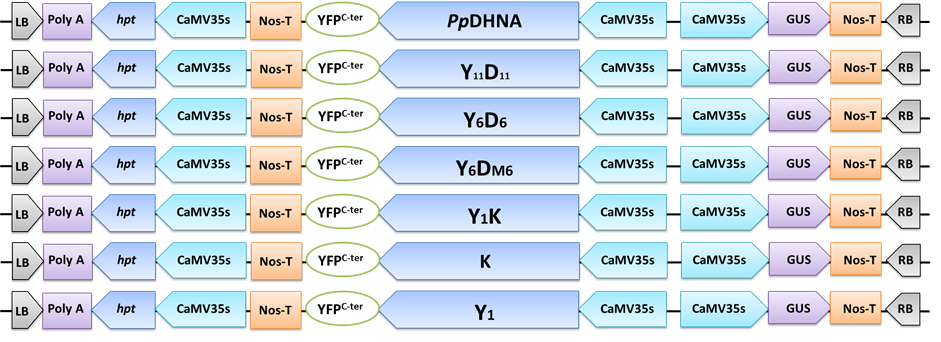
**

**S1****3 Figure.** Schematic representation of expression cassette in pCAMBIA1301 binary vector used for BiFC transient assays on onion leaf scales.
