# Supplementary material for: Multiple copies of a novel amphipathic α-helix forming segment in Physcomitrella patens dehydrin play a key role in abiotic stress mitigation
Source: J Biol Chem. 2021 Mar 26;296:100596. doi: 10.1016/j.jbc.2021.100596 (PMC8100072; doi:10.1016/j.jbc.2021.100596)

**Multiple copies of a novel amphipathic α-helix forming segment in *Physcomitrella patens* dehydrin play a key role in abiotic stress mitigation**

Gouranga Upadhyaya^1^, Arup Das^1^, Chandradeep Basu^2¶^, Tanushree Agarwal^1¶^, Chandra Basak^1^, Chandrima Chakraborty^1^, Tanmoy Halder^1^, Gautam Basu^2^, Sudipta Ray^1^*

**S1 Table**. Details of amino acid residues of different Y-segments occurring in PpDHNA protein with their corresponding amino acid position. The conserved Y residue is shown in red.

| **Y Segments** | **Position** (in aa) | **Sequence** |
| --- | --- | --- |
| Y_1_ | 22-27 | **DEYGNP** |
| Y_2_ | 57- 62 | **DNYGNP** |
| Y_3_ | 92-97 | **DSYGNR** |
| Y_4_ | 125-130 | **DNYGDR** |
| Y_5_ | 158-163 | **DNYGNR** |
| Y_6_ | 191-196 | **DSYGTR** |
| Y_7_ | 226-231 | **DNYGNP** |
| Y_8_ | 259-264 | **DNYGNR** |
| Y_9_ | 292-297 | **DNYGNP** |
| Y_10_ | 325-330 | **DSYGNP** |
| Y_11_ | 362-367 | **DTYGDR** |

**S2 Table**. Details of amino acid residues of the D and K-segment occurring in PpDHNA protein. The amino acid position of the corresponding D and K-segments occurring in PpDHNA are shown in the table. The conserved residues are shown in red. The calculated hydrophobicity and hydrophobic moment of D and K-segment were represented.

| **D- segment** | **Position**  (in aa) | **Sequence** | **Hydrophobicity**  <H> | **Hydrophobic moment** <µH> |
| --- | --- | --- | --- | --- |
| D_1_ | 30- 40 | ***EG*IM*D*KVKNAV** | 0.163 | 0.569 |
| D_2_ | 65-75 | ***EG*LV*D*KAKDAV** | 0.055 | 0.493 |
| D_3_ | 98-108 | ***EG*IV*D*RAKDAV** | 0.062 | 0.500 |
| D_4_ | 133-143 | ***EG*LA*D*RAKDAV** | -0.030 | 0.458 |
| D_5_ | 164-174 | ***EG*VV*D*KAKDAV** | 0.011 | 0.460 |
| D_6_ | 199-209 | ***EG*LV*D*RAKDAV** | 0.053 | 0.493 |
| D_7_ | 234-244 | ***EG*VV*D*RAKDAV** | 0.009 | 0.460 |
| D_8_ | 265-275 | ***EG*IV*D*KAKDAV** | 0.064 | 0.500 |
| D_9_ | 300-310 | ***EG*IV*D*RAKDAV** | 0.062 | 0.500 |
| D_10_ | 334-344 | ***EG*ML*D*KAKDDF** | 0.009 | 0.505 |
| D_11_ | 370-380 | ***EG*IG*D*KVRDAI** | 0.086 | 0.573 |
| K-segment | 542-552 | **KGIITKIKEKL** | 0.251 | 0.577 |

| **Name of Protein** | **Number of amino acid** | **Position**  **(in amino acid)** | **Molecular weight (KDa)** |
| --- | --- | --- | --- |
| PpDHNA | 554 aa | 1 to 554 | 59.16 KDa |
| Y_11_D_11_ | 421 aa | 1 to 421 | 44.87 KDa |
| Y_6_D_6_ | 222 aa | 1 to 222 | 23.58 KDa |
| Y_6_D_M6_ | 222 aa | 1 to 222 | 23.58 KDa |
| Y_1_K | 197 aa | [1 to 37] + [394 to 554] | 21.30 KDa |
| K | 160 aa | 394 to 554 | 17.16 KDa |
| Y_1_ | 37 aa | 1 to 37 | 4.15 KDa |

**S3 Table.** Compositional details of PpDHNA and its deletion mutants

**S4 Table.** Details of amino acid residues of shuffled D-segments present in the Y_6_D_M6_ deletion mutant. The hydrophobicity and hydrophobic moment of shuffled D segments were calculated and represented in the table.

| **D- segment** | **Position**  (in aa) | **Sequence** | **Hydrophobicity**  <H> | **Hydrophobic moment** <µH> |
| --- | --- | --- | --- | --- |
| D_M1_ | 30- 40 | **QMENKGDIKVA** | 0.032 | 0.016 |
| D_M2_ | 65-75 | **QVEDKGALKAD** | -0.076 | 0.037 |
| D_M3_ | 98-108 | **RVEDKGAIRAD** | -0.141 | 0.083 |
| D_M4_ | 133-143 | **AAEDRGHLKAD** | -0.129 | 0.065 |
| D_M5_ | 164-174 | **RVEDKGAVKAD** | -0.192 | 0.136 |
| D_M6_ | 199-209 | **QVEARGDLKAD** | -0.078 | 0.090 |

**S5 Table**. List of primers used in the amplification of the PpDHNA cds and the deletion mutants (Y_11_D_11_, Y_6_D_6_, Y_6_D_M6_, Y_1_K, Y_1_, K and Y) along with the primers for *hpt* gene. The sequence of the GFP and actin primers used for the qRT-PCR analysis are also provided. The amplicon size in each case is represented in the table.

| **Name** | **Primers** | **Primer sequence** | **Amplicon Size** (bp) |
| --- | --- | --- | --- |
| *PpDHNA* | Forward | 5’CATATGAATCAGTACGGAAGAGAACAGCAAG3’ | 1665 |
|  | Reverse | 5’CTCGAGGTGGTGCAGCTTCTCCTTGATCTTAGTTATG3’ |  |
| Y_11_D_11_ | Forward | 5’CATATGAATCAGTACGGAAGAGAACAGCAAG3’ | 1263 |
|  | Reverse | 5’CTAAGATCGAGGGCTCGTTCAC3’ |  |
| Y_6_D_6_ | Forward | 5’CATATGAATCAGTACGGAAGAGAACAGCAAG3’ | 666 |
|  | Reverse | 5’GCTGATTGTTATAGCCGGTG3’ |  |
| Y_6_D_M6_ | Forward | 5’CATATGAATCAGTACGGAAGAGAACAGCAAG3’ | 666 |
|  | Reverse | 5’GCTGATTGTTATAGCCGGTG3’ |  |
| Y_1_ | Forward | 5’CATATGAATCAGTACGGAAGAGAACAGCAAG3’ | 111 |
|  | Reverse | 5’CTCGAGCACCTTTTCCATTATACCCTC3’ |  |
| K | Forward | 5’ATGGACAAGGTGGGAACCGACGCTTACGTGCATG3’ | 486 |
|  | Reverse | 5’CTCGAGGTGGTGCAGCTTCTCCTTGATCTTAGTTATG3’ |  |
| Y_1_K | Forward | 5’CATATGAATCAGTACGGAAGAGAACAGCAAG 3’ | 594 |
|  | Reverse | 5’CTCGAGGTGGTGCAGCTTCTCCTTGATCTTAGTTATG3’ |  |
| Hygromycin (*hpt*) | Forward | 5’ATGAAAAAGCCTGAACTCACCGCGAC3’ | 1026 |
|  | Reverse | 5’TTCCTTTGCCCTCGGACGAGTGCTG3’ |  |
| *Actin*  (qRT-PCR) | Forward | 5’GCAACTGGGATGATATGGAG3’ | 114 |
|  | Reverse | 5’TAGCCTTCGGGTTAAGAGGT3’ |  |
| GFP  (qRT-PCR) | Forward | 5’GCACAAGCTGGAGTACAA3’ | 100 |
|  | Reverse | 5’GATGTTGTGGCGGATCTT3’ |  |

**S6 Table**. ^1^H and ^13^C Chemical shifts (ppm) of D_1_ in aqueous buffer at 277 K.


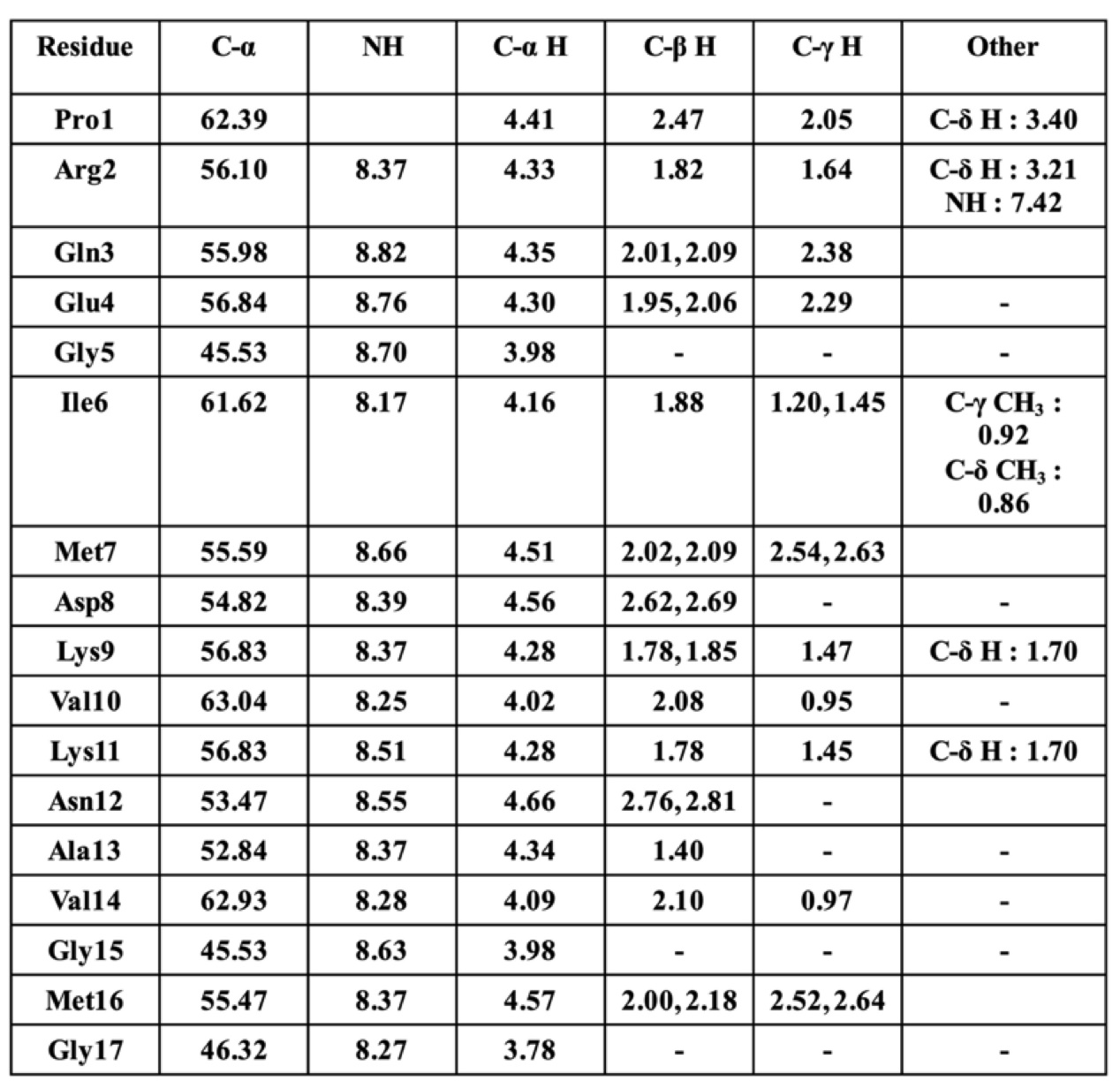


**S7 Table**. ^1^H and ^13^C Chemical shifts (ppm) of D_M1_ in aqueous buffer at 277 K.


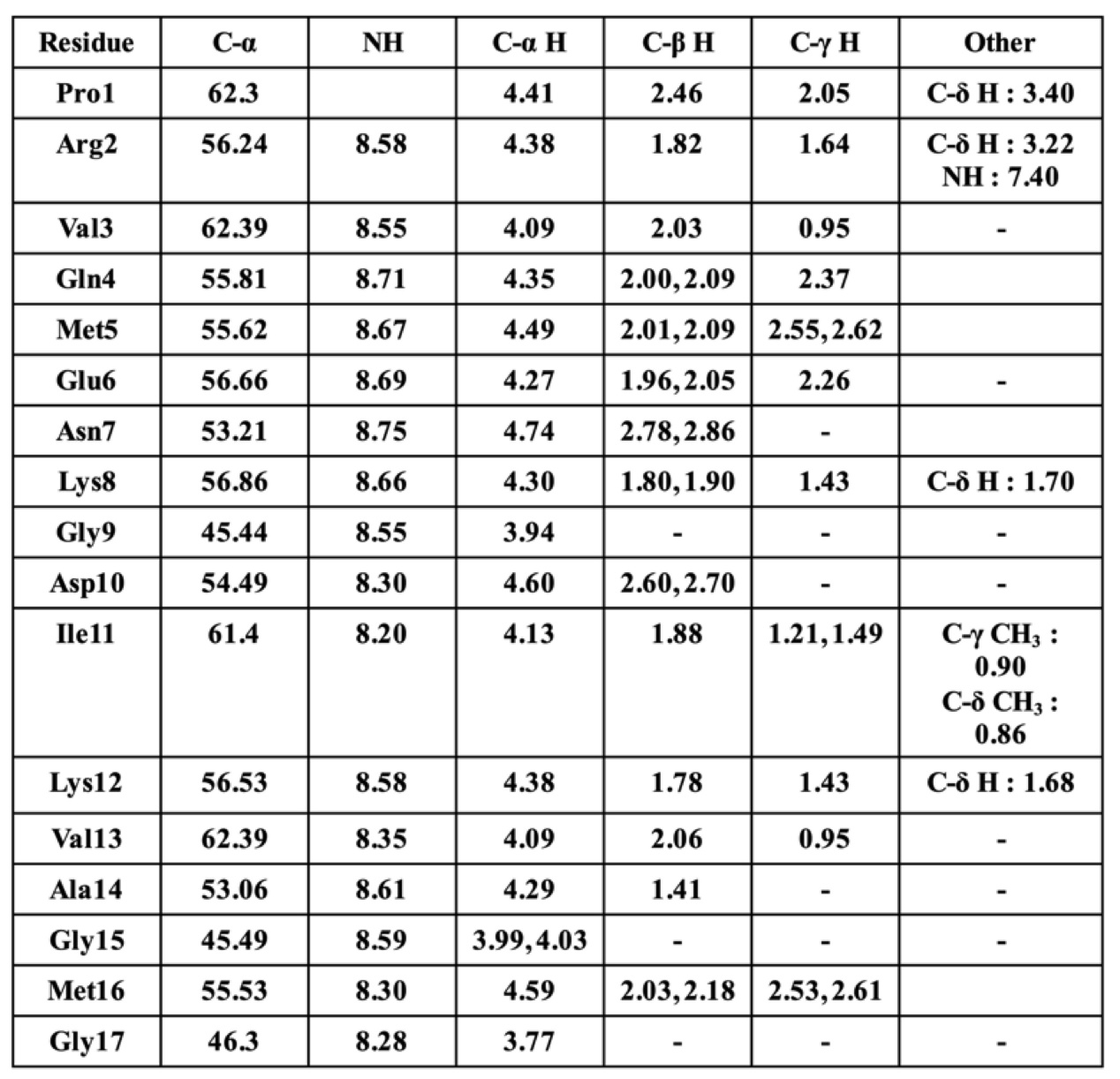

Supplement: Supplementary file 2 — Tables S1 to S7 [file mmc2.docx]
